# When Machine Learning Meets 2D Materials: A Review

**DOI:** 10.1002/advs.202305277

**Published:** 2024-01-26

**Authors:** Bin Lu, Yuze Xia, Yuqian Ren, Miaomiao Xie, Liguo Zhou, Giovanni Vinai, Simon A. Morton, Andrew T. S. Wee, Wilfred G. van der Wiel, Wen Zhang, Ping Kwan Johnny Wong

**Affiliations:** ^1^ ARTIST Lab for Artificial Electronic Materials and Technologies, School of Microelectronics Northwestern Polytechnical University Xi'an 710072 P. R. China; ^2^ Yangtze River Delta Research Institute of Northwestern Polytechnical University Taicang 215400 P. R. China; ^3^ Instituto Officina dei Materiali (IOM)‐CNR Laboratorio TASC Trieste I‐34149 Italy; ^4^ Advanced Light Source (ALS) Lawrence Berkeley National Laboratory Berkeley CA 94720 USA; ^5^ Department of Physics and Centre for Advanced 2D Materials (CA2DM) and Graphene Research Centre (GRC) National University of Singapore Singapore 117542 Singapore; ^6^ NanoElectronics Group, MESA+ Institute for Nanotechnology and BRAINS Center for Brain‐Inspired Nano Systems University of Twente Enschede 7500AE The Netherlands; ^7^ Institute of Physics University of Münster 48149 Münster Germany; ^8^ NPU Chongqing Technology Innovation Center Chongqing 400000 P. R. China

**Keywords:** 2D materials, data‐driven approach, machine learning

## Abstract

The availability of an ever‐expanding portfolio of 2D materials with rich internal degrees of freedom (spin, excitonic, valley, sublattice, and layer pseudospin) together with the unique ability to tailor heterostructures made layer by layer in a precisely chosen stacking sequence and relative crystallographic alignments, offers an unprecedented platform for realizing materials by design. However, the breadth of multi‐dimensional parameter space and massive data sets involved is emblematic of complex, resource‐intensive experimentation, which not only challenges the current state of the art but also renders exhaustive sampling untenable. To this end, machine learning, a very powerful data‐driven approach and subset of artificial intelligence, is a potential game‐changer, enabling a cheaper – yet more efficient – alternative to traditional computational strategies. It is also a new paradigm for autonomous experimentation for accelerated discovery and machine‐assisted design of functional 2D materials and heterostructures. Here, the study reviews the recent progress and challenges of such endeavors, and highlight various emerging opportunities in this frontier research area.

## Graphical abstract

The family of 2D materials is an unprecedented platform for materials by design, thanks to their ever‐expanding material portfolio with rich internal degrees of freedom. The study provides a comprehensive overview of the recent progress, challenges and emerging opportunities in a frontier research area that exploits machine learning—a very powerful data‐driven approach and subset of artificial intelligence—for 2D materials.

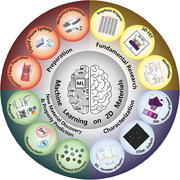

## 1 Introduction

Since the discovery of graphene, 2D materials have attracted much attention from researchers across the globe, with significant achievements in their preparation, characterization, theoretical analysis, and application. As with other fields, materials science has gone through a shift from the traditional paradigm of experimental science to the modern paradigm of data exploration.^[^ ^1^ ^]^ Against this backdrop, theoretical approaches, such as density functional theory (DFT) and molecular dynamics (MD), have been developed to analyze microstructures of materials and guide future research. However, due to the growing amount of data and limited computational resources, these methods are becoming increasingly time‐consuming. To overcome this challenge, a paradigm shift from ab initio calculations to extensive data exploration has occurred in materials science, with machine learning (ML) being an effective means of realizing this shift. ML encompasses a wide array of mathematical algorithms and model systems, such as deep learning (DL), deep neural network (DNN), and support vector machine (SVM) algorithms, etc. The goal of ML is to enable computers to learn from data, improve their performance on tasks, and make accurate predictions or decisions without explicit programming. There exists a large body of literature on ML algorithms, and more in‐depth discussions can be found elsewhere.^[^ ^2^, ^3^ ^]^ ML has been widely adopted across multiple disciplines. Especially, the successful application in high‐energy physics,^[^ ^4^ ^]^ drug design,^[^ ^5^ ^]^ medical diagnosis,^[^ ^6^ ^]^ chip design,^[^ ^7^ ^]^ and text recognition^[^ ^8^ ^]^ also accelerates the integration of different knowledge and domains. The advantages of ML over traditional methods in terms of recognition, search, and prediction tasks have provided novel solutions to various conundrums in a broad spectrum of disciplines, including the frontiers of 2D material sciences.

**Figure 1:**
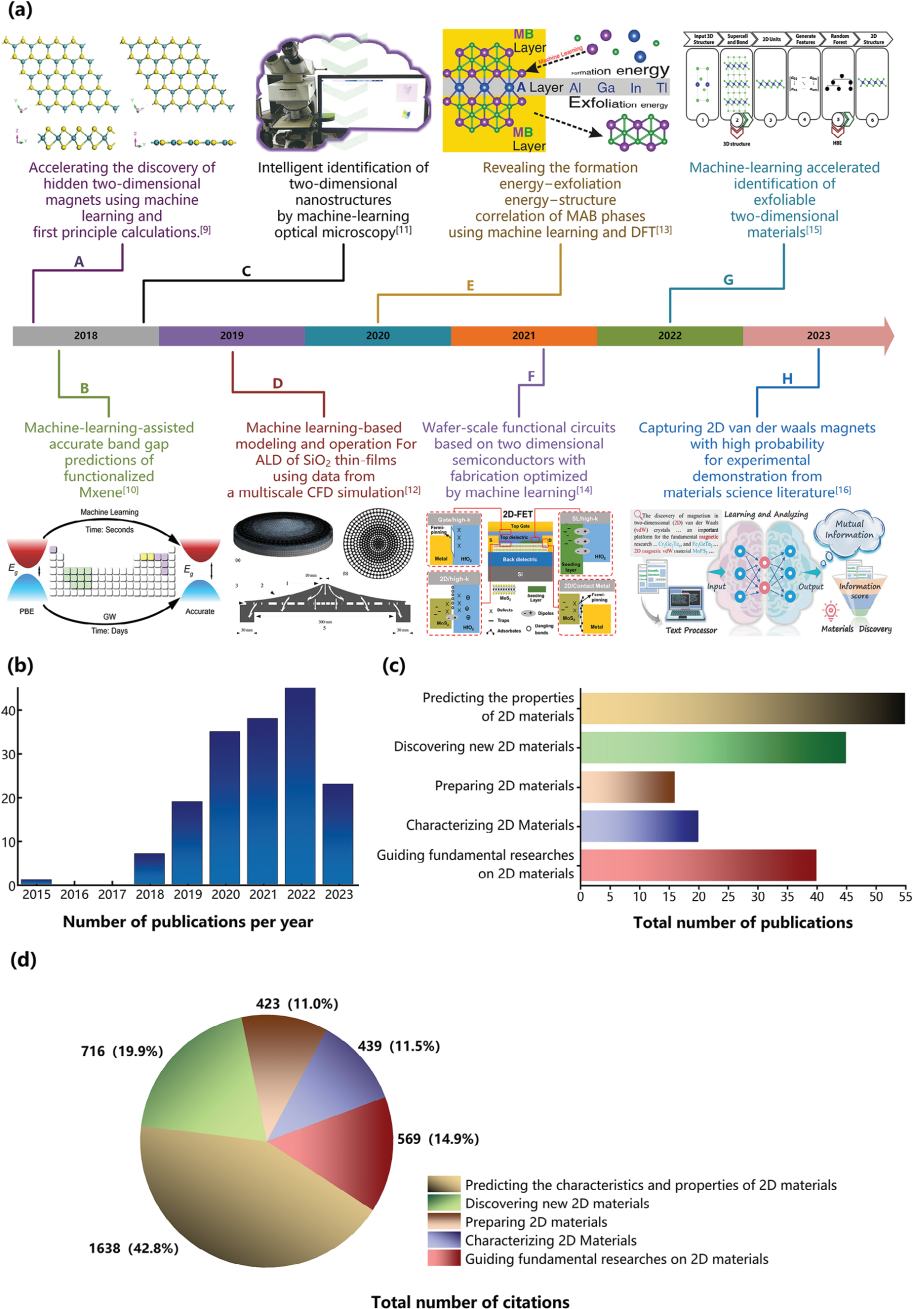
Advances in ML‐based research on 2D materials (as of July 2023). a) Major breakthroughs in 2D materials enabled by ML‐based approaches.^[^ ^9^ ^]^ Reproduced with permission.^[^ ^10^, ^11^, ^12^, ^13^, ^14^, ^15^, ^16^ ^]^ Copyright 2018, American Chemical Society; 2018, Springer Nature; 2019, Elsevier; 2020, American Chemical Society; 2021, Springer Nature; 2022, IOP Publishing; 2023, John Wiley and Sons. b) Number of publications of ML‐enabled studies on 2D materials. c) Number of publications of ML‐based research categorized based on five research directions. d) Number of citations in the five research directions (total citations > 3700).

**Figure** **1** presents a comprehensive analysis of 187 relevant publications by providing an overview of four types of information, including the major breakthroughs enabled by ML‐based approaches, the annual number of publications, the number of publications in specific research directions and the number of citations. Figure 1a displays several major breakthroughs with the involvement of ML. Since 2018, there has been a surge of ML‐based studies on 2D materials. In January 2018, Miyazato et al. employed a gaussian naive bayes classification algorithm to search for novel magnetic 2D materials.^[^ ^9^ ^]^ In May of the same year, Rajan et al. presented an ML model for bandgap predictions of functionalized MXenes.^[^ ^10^ ^]^ Subsequently, in August 2018, Lin et al. proposed a SVM algorithm for the characterization of graphene, MoS_2_ and their heterostructures, including the identification of the thickness and even the stacking order.^[^ ^11^ ^]^ In September 2019, for the first time, Ding et al. developed a multiscale data‐driven model to explore the application of ML in the preparation of 2D materials.^[^ ^12^ ^]^ ML has opened up more interesting applications in the field of 2D materials since 2020. In June 2020, Siriwardane et al. explored the correlation between the exfoliation energy, formation energy, and structural factors of layered ternary compounds with hexagonal and orthorhombic crystal symmetries using ML and DFT.^[^ ^13^ ^]^ Moreover, Chen et al.’s work, published in October 2021, illustrated the feasibility of using ML in device‐processing optimization for 2D materials.^[^ ^14^ ^]^ In November 2022, Vahdat et al. developed a ML approach to evaluate the exfoliation potential of 3D compounds into 2D layers.^[^ ^15^ ^]^ Most recently, in January 2023, Song et al. established a framework that enables the identification of 2D van der Waals (vdW) magnets with high probability for experimental verification, based on a large body of literature in materials science.^[^ ^16^ ^]^ As shown in Figure 1b, the number of published articles per year related to ML in 2D materials research is increasing steadily, and this trend is likely to continue. To provide further insight in ML‐enabled advances, Figure 1c categorizes the publications based on the prediction, discovery, preparation, characterization, and fundamental research of 2D materials. Figure 1d presents the statistics of the article citations according to these five research directions, with a total amount of citations in excess of 3700. These findings suggest that the intersection of ML and 2D materials is rapidly gaining recognition as a prominent field, with the active implementation of ML techniques anticipated to expedite the development of 2D materials.

At present, ML has seen increasing popularity in 2D materials, with research directions expanding from merely discovering and predicting properties to preparing, characterizing, and exploring new physical phenomena. In studies related to material properties, ML has been combined with DFT and MD to explore the thermal properties,^[^ ^17^, ^33^, ^34^, ^35^, ^36^, ^37^, ^38^, ^46^ ^]^ bandgaps,^[^ ^47^, ^48^, ^49^, ^50^, ^51^, ^52^, ^53^, ^54^, ^55^ ^]^ and mechanical properties^[^ ^56^, ^57^, ^58^, ^59^ ^]^ of various materials, thereby accelerating the pace of research in this field. Furthermore, ML has also been utilized for the discovery of novel 2D materials, including catalytic,^[^ ^60^, ^61^, ^62^, ^63^, ^64^, ^65^, ^66^, ^67^, ^68^ ^]^ photoelectric,^[^ ^69^, ^70^, ^71^, ^72^, ^73^, ^74^, ^75^, ^76^, ^77^ ^]^ and magnetic materials.^[^ ^78^, ^79^, ^80^, ^81^, ^82^, ^83^, ^84^, ^85^, ^90^, ^91^, ^92^ ^]^ In terms of preparing 2D materials, ML methods have been applied to deposition and exfoliation to enable easier and more controllable preparation of 2D materials such as WTe_2_,^[^ ^93^ ^]^ MoS_2_,^[^ ^94^, ^95^, ^96^ ^]^ and WS_2_.^[^ ^97^, ^98^ ^]^ When it comes to characterizing 2D materials, ML has been combined with characterization techniques, like Raman spectroscopy, transmission electron microscopy, optical microscopy, and imaging, to acquire accurate information such as the thickness^[^ ^99^, ^100^, ^101^, ^102^, ^103^, ^104^, ^105^, ^106^, ^107^, ^108^, ^109^, ^110^, ^111^, ^112^, ^113^ ^]^ and defects^[^ ^114^, ^115^, ^116^, ^117^ ^]^ of materials. The utilization of ML in 2D materials research, along with the algorithmic processing of experimental data, has the potential to support data analysis, leading to more conclusive results on the reliability and reproducibility of given datasets. Moreover, ML can facilitate the systematic correlation of material structure and properties, potentially guiding the discovery of new 2D materials.

To our knowledge, there have been seven published reviews related to the current topic,^[^ ^1^, ^2^, ^118^, ^119^, ^120^, ^121^, ^122^ ^]^ each covering some specific aspects of it. However, a systematic overview with updated references is highly desirable. This review aims to explore ML‐enabled studies on the preparation and characterization of 2D materials and theoretical analysis, providing a detailed summary of existing publications in this regard. We focus on the advances of ML‐based research in terms of predicting the properties of 2D materials as well as guiding to the discovery, preparation, and characterization of new 2D material systems; popular ML algorithms, descriptors, and workflows are also introduced. In Section 2, we introduce the general computing process of ML and some popular ML algorithms adopted for result optimization. In Section 3, we review the advances in the ML‐enabled prediction of the properties of 2D materials, including energy characteristics and thermal, electronic, and mechanical properties. Section 4 covers the role of ML in the discovery of new materials such as catalytic, photoelectric, and magnetic materials. Section 5 focuses on the ML‐enabled optimization of preparation techniques of 2D materials. In Section 6, we present the advantages of using ML algorithms in the characterization of 2D materials, examples of which include identifying layers of materials, and locating and classifying defects. Section 7 provides a summary of ML‐based fundamental research on 2D materials and other emerging directions. Finally, in Section 8, we outline the current challenges and future prospects of ML‐based studies on 2D materials.

## 2 Machine‐Learning Algorithms

**Figure 2:**
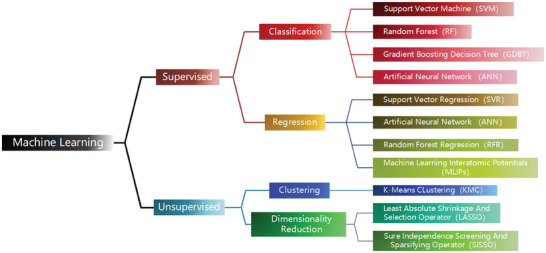
ML algorithms applied to 2D materials research.^[^ ^124^ ^]^

**Figure 3:**
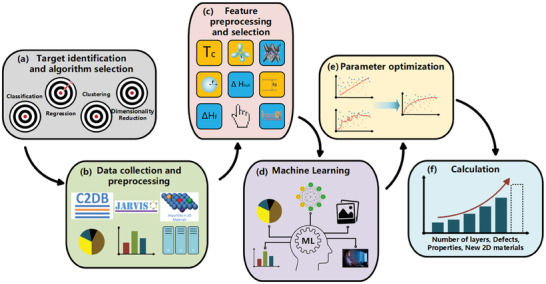
ML model construction workflow: a) turn real‐world problems into mathematical tasks, identify the type of available data and the target task (classification, regression, clustering); b) collect and preprocess data, the quantity and quality of the data will determine the model's performance; c) preprocess and select features, visualize data analysis results, find potential correlations between variables, assess whether the data are balanced, and partition data into a training set and a test set; d) select and train the ML model; e) assess the parameters, analyze modeling errors, and fine‐tune parameters for optimization; f) output the prediction results.

ML algorithms applied to 2D materials research can either be supervised or unsupervised, as illustrated in **Figure** **2**. Supervised learning algorithms can be categorized into either classification or regression ones, depending on whether the measured value is discrete or continuous.^[^ ^123^ ^]^ Unsupervised learning algorithms, depending on their purpose, can be divided into clustering and dimensionality reduction algorithms. As elaborated in **Figure** **3**, the construction of an ML model generally involves six steps and will be discussed in detail in the following sections.

### 2.1 Target Identification

There is no “one‐size‐fits‐all” solution for solving problems related to 2D materials. The choice of ML algorithm will vary depending on the specific research target. Therefore, identifying the target and selecting a proper algorithm are essential. Supervised learning algorithms deduce function from labeled training data, where the function connects the known input‐unknown output pairs. Among them, regression algorithms can output specific numerical values and are effective for predicting the properties of 2D materials, such as bandgap^[^ ^47^ ^]^ and Curie temperature (*T_C_*),^[^ ^140^ ^]^ etc. On the other hand, classification algorithms have significant advantages in solving discrete tasks and answering yes/no questions, such as the prediction of thermodynamic stability^[^ ^17^ ^]^ or magnetic properties^[^ ^137^ ^]^, etc.

Contrary to supervised learning algorithms, unsupervised learning algorithms, which encompass clustering and dimension reduction, deal with unlabeled data and aim to seek and deduce potential connections among samples. K‐means clustering (KMC), the most commonly used clustering algorithm in 2D materials research, swiftly and automatically groups numerous 2D materials into clusters with similar features, thereby unveiling the characteristics of these unlabeled clusters. For example, in studies involving identification of the number of layers in 2D materials, distinct RGB values are linked to various layer numbers, allowing materials with similar or identical layer numbers to be grouped into the same cluster.^[^ ^106^ ^]^ Dimension reduction algorithms, such as least absolute shrinkage and selection operator (LASSO), are primarily utilized to map data from the original high‐dimensional space to the low‐dimensional space, thereby reducing model complexity and improving generalization performance. In addition, two ML algorithms that are not commonly employed in 2D material studies are semi‐supervised learning and reinforcement learning. Semi‐supervised learning, which combines with characteristics from both supervised and unsupervised learning, is utilized in scenarios of incomplete data labeling, effectively reducing the high costs associated with the process. For example, in study of discovering new vdW magnets, semi‐supervised learning is applied to cope with the challenges of sparsely labeled materials data, thereby enhancing the performance of ML models.^[^ ^90^ ^]^ Reinforcement learning (RL), unlike the algorithms mentioned above, does not rely on a pre‐existing dataset to train models. Instead, it relies on the interaction between an agent and an environment to learn the optimal strategy through trial and error. Such algorithms are covered in the fundamental research on 2D materials summarized in Section 7, including tasks like finding the optimal design of the MoS_2_’s Kirigami structure.^[^ ^201^ ^]^

It is essential to acknowledge that, in many instances, directly determining the most appropriate ML algorithm for a specific task in 2D materials research can be challenging. In practice, it is common to utilize a range of ML algorithms to train multiple models tailored to a specific task. Subsequently, these models undergo thorough comparison, and the one demonstrating the lowest error and uncertainty is selected.

### 2.2 Data Collection and Preprocessing

Building a database is the next step after selecting the algorithm. The quality and quantity of the data, which serve as input, are crucial factors that will determine the model's reliability and performance. The desired data can be collected from several sources, mainly including published articles, experiments, as well as computations. Collecting data from publications allows access to large amounts of data, but ensuring the quality and reliability of the data may pose particular challenges in terms of data uncertainty assessment and intricate data preprocessing.^[^ ^18^ ^]^ Furthermore, acquiring significant volumes of research data under limited experimental conditions is by itself time‐consuming, and the measured results typically come from a small sample set. On the other hand, first‐principles calculations have mitigated the limitations of experiments, enabling a substantial amount of available data to be generated in a relatively shorter time and with a lower cost. Nevertheless, only with unified first‐principles calculation parameter standards can this method become one of the preferred strategies for constructing material databases.^[^ ^19^ ^]^ In this regard, Torelli et al. utilized first‐principles calculations with the PBE+U functional to screen for magnetic insulators based on the C2DB, which accurately handles strongly correlated systems, in contrast to all computations in C2DB that are performed using PBE functions. In addition to the known compounds within the database, they also constructed and discovered 17 novel insulating magnetic materials with a crystal structure based on CrBrS. Finally, they identified and predicted 10 candidates with critical temperatures surpassing that of CrI_3_. It is noteworthy that the calculated exchange constants for ferromagnetic materials in this study have been integrated into the C2DB.^[^ ^20^ ^]^ The another method is to obtain substantial volumes of data from open‐source databases available on repository websites. One of the earliest open‐source databases, the Inorganic Crystal Structure Database (ICSD), was created in 2002 and comprehensively covers crystal structure information for non‐organic compounds obtained from previous calculations and experiments.^[^ ^21^ ^]^ The Materials Project database, founded in 2013 and built upon the ICSD,^[^ ^22^ ^]^ stands as the core program of the Materials Genome Initiative,^[^ ^23^ ^]^ employing high‐throughput calculations to unveil the properties of all known inorganic materials. The Computational 2D Materials Database (C2DB), established in 2018 and constructed using high‐throughput calculations, is one of the most widely utilized open‐source databases, encompassing ≈4000 types of 2D materials.^[^ ^24^ ^]^ It is essential to acknowledge that the continuous emergence of these extensive open‐source databases has supplied an ample amount of high‐quality and easily accessible data for the training of ML models. For instance, publications that predict the properties of 2D materials and develop new 2D materials frequently employ open‐source databases. **Table** **1** lists several other popular open‐source databases used for research on 2D materials.

**Table 1:** Popular open‐source databases of 2D materials.

Database	URL
Computational 2D Materials Database (C2DB)	https://www.cmr.fysik.dtu.dk/c2db/c2db.html
aNANt	https://www.anant.mrc.iisc.ac.in
Materials Project	https://www.materialsproject.org
JARVIS‐DFT	https://www.jarvis.nist.gov/jarvisdft
Materials Cloud	https://www.materialscloud.org/discover
2D Materials Encyclopedia (2DMatPedia)	https://www.2dmatpedia.org
2D Materials	https://www.materialsweb.org/twodmaterials
Inorganic Crystal Structure Database (ICSD)	https://www.2fiz‐karlsruhe.de/icsd_home.html
Crystallography Open Database (COD)	https://www.crystallography.net
Cambridge Structural Database (CSD)	https://www.ccdc.cam.ac.uk/structures
Aflow	https://www.aflowlib.org
Open Quantum Materials Database (OQMD)	https://www.oqmd.org

In certain cases, it is necessary to extract effective data from multiple material databases and consolidate the data into another database to fulfill the data volume required for ML model training. For instance, to identify non‐magnetic 2D semiconducting materials with hole‐induced ferromagnetism, Meng et al. collected 2D crystal structures from three databases: 2D Materials Encyclopedia (2DmatPedia), C2DB, and Materials Cloud. In the data extraction process, they employed a high‐throughput screening method to exclude magnetic metals, along with filtering out repeated structures and those with low thermodynamic stability, resulting in the selection of 3000 materials for subsequent hole doping simulations.^[^ ^92^ ^]^ When left unprocessed, this extracted data, especially data not sourced from open‐source databases, may pose challenges in analysis and even become unusable due to missing values, noise, and inconsistencies. Therefore, it is essential to address these issues by manually filling in missing values, employing regression or clustering algorithms to reduce noise, utilizing clustering algorithms to identify and reduce outliers, and transforming the data into a uniform format.^[^ ^25^ ^]^ In the study utilizing ML algorithms to identify the number of graphene layers from optical microscope (OM) images, experimental uncertainties like non‐uniform illumination and camera sensor degradation over time can render the microscope‐captured images unsuitable for direct identification. Therefore, Yang et al. applied a median filter algorithm to reduce noise, and then modeled the background's color profile using a polynomial function, following by subtracting it from the original image. Their approach resulted in an improved image quality and uniformity.^[^ ^110^ ^]^ Subsequently, the preprocessed data is typically partitioned into three subsets: a training set, a validation set, and a test set, to prepare for the subsequent training of ML models. The training set is used to train and fit the model during the learning process. Following the training of ML models on this set, validation sets are utilized for cross‐validation to assess the accuracy of the model and adjust the hyperparameters. The testing set is employed to assess the model's accuracy, with the labels of the testing set concealed during the evaluation process. The model's predictions are then compared to the actual values to evaluate its generalization capacity.

### 2.3 Feature Engineering

Another crucial factor influencing the performance of ML models is feature engineering, which aims to remove redundant features and establish proper structure‐property relationships. Features (also known as descriptors) describe the properties of materials, obtained by extracting object attributes from prepared data and converting them into numerical or categorical formats. In feature selection, different parameters can be used as features for chemical and material structures, such as stoichiometric properties (fraction and number of elements presented, etc.), elemental properties (range of atomic radii and average atomic number, etc.), electronic properties (bandgap, dielectric constant, work function, electron density, and electron affinity, etc.), and crystal features (translation vectors, fractional coordinates of atoms, radial distribution functions, and Voronoi tessellations of atomic positions, etc.).

When selecting features to describe a material, it is important to consider its physical properties, particularly, the periodicity and invariance.^[^ ^26^ ^]^ Initial attempts to create material descriptors relied solely on chemical composition, such as bond lengths, bond angles, etc. The functional forms employed for constructing interatomic potentials and fitting potential energy surfaces depend on the components of a meticulously selected representation of atomic neighborhoods.^[^ ^27^ ^]^ Hansen et al. employed the pairwise interatomic force fields method to estimate atomization and total energies of molecules, ensuring both symmetry and invariance in describing materials, while also proving its effectiveness in conducting preliminary stability assessments of equilibrium geometries.^[^ ^28^ ^]^ While the performance of pairwise potentials is already quite good, their performance for out‐of‐equilibrium molecular geometries is strongly degraded. Therefore, simple metrics were subsequently incorporated to encode crystal structures, with the goal of maximizing the predictive capabilities of the ML models. At the core of this method is the ability to encode diverse material structures into computer‐interpretable descriptors while satisfying the periodic conditions of materials. This encompasses four representative structural features: the structure graph, Coulomb matrix, topological descriptor, and diffraction fingerprint.^[^ ^29^ ^]^ Hansen et al. also introduced the Bag of Bonds model, a distinctive variant of the Coulomb matrix, which employs a vectorized representation of molecules to efficiently capture substantial non‐locality within chemical space and proficiently describe collective interactions among numerous atoms or bonds. This representation maintains natural invariance under molecular rotations and translations.^[^ ^28^ ^]^ This study only can map from structure to properties but lacks the capability for reverse map from target properties to atomic structure, thereby hindering the realization of the reverse material design paradigm.^[^ ^30^ ^]^ Xie et al. employed a crystal graph to represent periodic crystal structures, capturing both atomic details and bonding interactions between atoms. They proceed to build a convolutional neural network model, called a crystal diffusion variational autoencoder, on this graph. This approach enables the direct learning of material properties from the atomic connections within the crystal and avoiding the need for an invertible representation.^[^ ^31^ ^]^

Another important consideration concerns the quantity and quality of features. Generally, a higher ratio of sample size to feature dimension tends to result in better model performance, while a lower ratio can result in longer model training times, increased computational overhead, and even potential overfitting. The most popular algorithm for feature selection in studies on 2D materials is the least absolute shrinkage and selection operator (LASSO) method. By establishing a penalty function, LASSO compresses the coefficient of some features and sets the regression coefficient of some features to zero in order to identify the features that have the strongest influence on the target material's properties.^[^ ^26^ ^]^ Additionally, Principal Component Analysis (PCA) and the Decision Tree (DT) method can also be employed for feature selection. In the study of identifying metallic transition‐metal dichalcogenides for hydrogen evolution, the feature selection methods, such as PCA and gradient boosting, are used to filter out the most significant features influencing hydrogen adsorption strength.^[^ ^64^ ^]^ In another study, the DT model, adept at capturing intricate data relationships while mitigating variance, is also employed to screen the important features for magnetic materials.^[^ ^139^ ^]^ When the existing features lack sufficient valid information to achieve satisfactory model performance, new features can be constructed based on domain knowledge or generated using algorithms like the Sure Independence Screening Sparsifying Operator (SISSO). The SISSO algorithm is designed to construct models by continuously integrating essential existing features, from which it identifies the optimal mathematical expressions for describing the relationships within the data. This approach also enhances the feasibility of achieving more interpretable materials design.^[^ ^17^ ^]^ Conversely, using numerous correlated features will increase feature dimensionality, which can prolong model training times and potentially lead to overfitting issues. In this case, we need to further either remove features with low correlation Coefficients with the target property or consider reconstructing features using the algorithms mentioned above.

### 2.4 Model Training

After selecting databases and features, the ML model is then trained, and its parameters are fine‐tuned so that it will reach more accurate predictions. Meanwhile, the hyperparameters of a ML model should be pre‐set and continually adjusted through various available methods, such as grid or random search, evolution strategies, Bayesian optimization, Hyperband, and racing.^[^ ^32^ ^]^ Following the model training, model validation and evaluation are usually performed to evaluate whether the trained model can accurately predict the target properties. Model validation involves sensitivity analysis and fitting degree analysis. Overlearning of a model can reduce its generalization capacity and lead to overfitting, where excessive consideration of details, including noise and normal errors, occurs. In contrast, if a model lacks an ability to map the relationship between data and show the complexity of feature, underfitting can occur. For these reasons, model validation is an important step. Cross‐validation is a popular method of validation when it comes to research on 2D materials. During cross‐validation, datasets are partitioned into several mutually exclusive subsets (also called “folds”), where (*k*‐1) subsets are used as the training set and the remaining subset as the validation set. The model is trained for *k* times, and the average value of the *k* results is returned. This process is called *k*‐fold cross‐validation.^[^ ^3^ ^]^

Model evaluation refers to the evaluation of a model's generalization capacity. Common evaluation indicators for regression models include mean squared error (MSE), mean absolute error (MAE), root mean square error (RMSE), and R‐Square (R^2^). MAE indicates the average difference of the predicted value from the actual value; MSE is the ratio of the squared errors between the actual values and estimated values to the times of estimation, which is a measure of changes in the data. The accuracy of the prediction model in describing the sample data increases as the MSE decreases. RMSE, the square root of MSE, is preferred in nonlinear fitting. R^2^, also known as the coefficient of determination, reflects the ability of a regression model to fit data, with a range from 0 to 1. A value closer to 1 indicates a better fit of the model to the data. It is important to note that R^2^ is a relative measure and can vary greatly among different models built on different datasets, with larger datasets having narrower distribution ranges generally resulting in higher R^2^ values. Therefore, R^2^ alone is not sufficient for model evaluation and should be considered along with other parameters. As for classification models, commonly used evaluation indicators include accuracy, precision (P), recall (R), F1 score, receiver operating characteristic curve (ROC), and area under the curve (AUC). Accuracy is the most basic evaluation indicator, calculated as the ratio of correctly classified samples to total number of samples. In many cases, accuracy alone cannot reflect the true performance of a model, so P and R are introduced to complement its limitations. P represents the probability of true positive predictions among all predicted positives, while R represents the probability of true positive predictions among all actual positives. P and R provide subjective and objective evaluations of a model's predictive ability, but they alone cannot comprehensively assess a model. Therefore, there is a need for an evaluation metric that takes both P and R into account, with the most common approach being the use of the F‐score. The F‐score is the weighted harmonic mean of P and R, and when their weights are equal, it becomes the commonly used F1 score, where a higher value indicates superior model performance. In addition, ROC is a curve that reflects the predictive performance of a model, while AUC represents the area under the ROC curve, ranging from 0.5 to 1. When evaluating a model's performance across different thresholds or dealing with sample imbalance, AUC and ROC serve as more meaningful evaluation metrics. A steeper ROC curve and a larger AUC are generally considered as indicators of better predictive capability of a model.

## 3 Predicting the Properties of 2D Materials

2D materials typically have more unique properties than their 3D parent materials and offer many possibilities for application. However, predicting their properties using conventional theoretical and computational methods is resource‐intensive. In this regard, ML offers an effective solution for studying 2D materials and accelerating their discovery. Popular ML models for property prediction include artificial neural networks (ANN)^[^ ^47^ ^]^, machine‐learning interatomic potentials (MLIPs)^[^ ^34^, ^125^ ^]^ and other regression models. For MLIPs algorithm, the interatomic potential generated by training an ML model on a large training database, which often involves thousands of DFT calculations, can predict target properties or attributes with better accuracy and speed than standard DFT calculations. In addition, Algorithms such as SISSO^[^ ^17^ ^]^ and LASSO^[^ ^33^, ^50^, ^51^, ^52^, ^53^ ^]^ are usually used to find the descriptor with the largest contribution to the target property, thereby optimizing the model's performance.

The fundamental concept of using ML for property prediction is to analyze and discover the nonlinear relationships between properties and related factors based on existing information, which can enhance our understanding of the underlying physical or chemical mechanisms. For instance, the ML model proposed by Garg et al. unveiled the explicit mathematical relationship between the shear modulus of graphene sheets and parameters such as aspect ratio, temperature, number of atomic planes, and the presence of defects.^[^ ^56^ ^]^ The generation of extensive theoretical results covering various properties can also contribute to enriching open‐source databases, thereby facilitating the discovery of materials with desired properties. In Thygesen et al.’s study, the ML model predicted 700 band structures of 2D semiconductors, which have been published on the C2DB web page.^[^ ^127^ ^]^ In addition, most ML algorithms used to predict various properties are now open‐source. A highly promising strategy involves integrating existing ML models and subsequently screening a wide range of materials tailored to specific applications, such as thermally stable magnetic semiconductor materials. This process is then followed by the evaluation of the most promising candidates through DFT calculations or experiments. Based on databases, such as C2DB and Materials Cloud, Dutta et al. trained ML models to classify materials as magnetic or non‐magnetic, and predict magnetic moment and anisotropy energy per metal atom for the members in the magnetic class. Using these predicted results, they proceeded to design 278 new mixed 3d‐5d transition metal compounds that could potentially exhibit both high magnetic moments and anisotropy energies through element replacements within the unit cell. After filtering through the ML model and verifying with DFT calculations, they identified 7 new materials with stability, significant magnetic moments, and substantial anisotropy energy.^[^ ^91^ ^]^ However, given the excessively complex and unique relationship between target properties and features, it is essential to conduct a detailed analysis of each property of 2D materials individually. The following sections introduce some of the advances made by ML‐based predictions of a wide range of 2D material properties (**Table** **2**), such as thermal stability,^[^ ^17^ ^]^ thermal conductivity,^[^ ^33^, ^34^, ^35^, ^36^, ^37^, ^38^, ^39^, ^40^, ^41^, ^42^, ^43^ ^]^ thermal expansion,^[^ ^44^, ^45^, ^46^ ^]^ energy band structure,^[^ ^127^, ^128^, ^129^ ^]^ bandgap,^[^ ^10^, ^47^, ^48^, ^49^, ^50^, ^51^, ^52^, ^53^, ^54^, ^55^ ^]^ shear modulus,^[^ ^56^ ^]^ fracture toughness,^[^ ^57^, ^58^, ^59^ ^]^ exfoliation energy,^[^ ^130^ ^]^ binding energy,^[^ ^131^, ^132^ ^]^ adsorption energy,^[^ ^133^, ^134^, ^135^, ^136^ ^]^ magnetic properties,^[^ ^137^, ^138^, ^139^ ^]^ *T_C_* ^[^ ^140^, ^141^ ^]^ and electrical breakdown limits,^[^ ^142^ ^]^ etc.

**Table 2:** A summary of ML‐based predictions of 2D material properties (Full names of the ML algorithms are listed in Appendix).

Properties	Data source	Materials	Source of descriptors	ML algorithms	Assessment metrics	Guiding suggestion	Ref.
Thermodyna‐mic stability	C2DB	Non‐magnetic materials	Atomic and structural properties	GBDT	RMSE = 0.165 R^2^ = 0.900	The stability condition includes formation energies below 0.0 eV atom^−1^ and energies above the convex hull ranging from 0.1 to 0.2 eV atom^−1^.	[17]
Thermal conductivity	MD simulation	WSe_2_	Lattice parameters, equations of state, cohesive energies, phonon dispersion, and elastic properties	GA		Thermal conductivity is higher along the armchair direction than zigzag due to phonon–phonon Umpklapp scattering.	[33]
	DFT calculation	Single‐layer penta‐graphene, phosphorene, silicene, MoS_2_, F‐diamane	Moment tensor	MLIPs		The ZA acoustic mode significantly contributes to thermal conductivity.	[34]
	MD simulation	CoSb_3_	Moment tensor	MLIPs		Phonon scattering properties are critical for calculating thermal conductivity.	[35]
	MD simulation	Graphene, phagraphene, haeckelite, C_2_N, C_3_N, C_7_N_6_, MoS_2_, penta‐SiP_2_	Moment tensor	MLIPs		Acoustic modes are the primary heat carriers in graphene.	[36]
	MD simulation	Graphene, C_2_N, MoS_2_, borophene, phosphorene, silicene, C_3_N	Moment tensor	MLIPs		Phonons are highly correlated with thermal conductivity.	[37]
	MD simulation	BeN_4_, MgN_4_, PtN_4_	Moment tensor	MLIPs		Thermal conductivity is higher in the armchair direction than in the zigzag direction due to differences in phonon group velocities.	[38]
	DFT calculation	MoS_2_, MoSe_2_, MoS_2(1−x)_Se_2x_	Bispectrum coefficients	MLIPs		The low thermal conductivity of the alloy is likely due to middle‐to‐high frequency phonons.	[39]
	DFT calculation	BC_2_N	Moment tensor	MLIPs		ZA mode is the dominant heat carrier.	[40]
	DFT calculation	Buckled silicene, graphene,and h‐XN (X = Ga, Al, and B)	Gaussian approximation potentials	MLIPs		Phonon‐phonon interactions are pivotal in determining lattice thermal conductivity.	[41]
	DFT calculation	2D substrate interfaces	ZA phonon bandwidth, ZA phonon resonance gap, vdW spring coupling constant, etc.	GPR		Polymethyl methacrylate, SiO_2_, HfO_2_, and Ge are the top‐performing substrate materials.	[42]
	C2DB	2D materials	Atomic mass, atomic number, covalent radius, number of valence electrons, pauli electronegativity, etc.	SISSO			[43]
Thermal expansion	MD simulation	Graphene, haeckelite, phagraphene, penta‐graphene and binary systems of C_3_N, BC_3_, C_2_N, C_7_N_6_, h‐BN	Moment tensor	MLIPs		Out‐of‐plane ZA mode vibrations are the primary cause of negative TEC.	[44]
	MD simulation	Graphene, phagraphene, C_3_N, BC_3_	Moment tensor	MLIPs		The substrate can reduce the formation of out‐of‐plane wrinkles, impacting the TEC value.	[45]
	MD simulation	Graphene	Atomic structures	ANN based MLIPs		TEC is governed by two competing mechanisms: rippling effect and thermal strain.	[46]
Band structure	C2DB	2D materials	radially decomposed projected density of states and energy decomposed operator matrix elements	GBDT	MAE = 0.150 RMSE = 0.140		[127]
	Self‐generated	WSe_2_	Band‐mapping data	ML			[128]
	Self‐generated	2D and 3D photonic crystals	Geometric dimensions, dielectric constants	ANN	MSE = 2.7 × 10^−6^		[129]
Bandgap	DFT calculation	Hybridized boron–nitrogen graphene	Atomic and structural properties	CNN	MAE = 0.090 RMSE = 0.120 R^2^ = 0.664	The bandgaps increase correspondingly as the concentration of BN increases.	[47]
	C2DB	2D materials	Dosef, Fmax, Smax,Masses, Energy, Hform, Cellarea, Volume, Gap_nosoc	SVR, MLP, GBDT, and RF	GBDT MAE = 0.030 RMSE = 0.090 R^2^ = 0.980	Adding Gap_nosoc to the feature space improves ML model performance.	[48]
	DFT calculation	MXenes	Boiling point and atomic radii, phases, melting points, bond lengths	KRR, SVR, and GPR	GPR MAE = 0.160 RMSE = 0.140 R^2^ = 0.830	Bandgap exhibits a strong positive correlation with bonding in elemental solids.	[10]
	DFT calculation	2D semiconductors	PBE results, valence‐band maximum, conduction‐band minimum, some elemental signatures, etc.	SVR, LR, and RFR	SVR RMSE = 0.080		[49]
	C2DB, 2DMatPedia	AB‐type materials	Melting point of element A, the minimum atom number, and the heat of fusion of element A, etc.	LASSO	MAE = 0.120 R^2^ = 0.970		[50]
	DFT calculation	2D hybrid organic–inorganic perovskites: A_2_BX_4_	Atomic weight of the ion at the X and B site ion, Moran's autocorrelation, Sanderson's electronegativities, etc.	RT, ERT, SVM, GPR, and ANN	ANN MSE = 0.010 MAE = 0.070 RMSE = 0.090 R^2^ = 0.980	The atomic weight of the ion at the X site is negatively correlated with the bandgap, while the B site ion exhibits the opposite effect.	[51]
	DFT calculation	Hybrid organic−inorganic materials with a 2D perovskite‐like crystal structure	Atomic and structural properties	XGBoost	R^2^ = 0.930	The bandgaps decrease with an increasing number of inorganic layers and bond angles.	[52]
	DFT calculation	Layered hybrid vdW heterostructures materials	Atomic and structural properties	FFNN, RVM, SVM, and RF	FFNN MSE = 0.016 R^2^ = 0.880		[53]
	DFT calculation	Layered 2D TMD vdW heterostructures	The sum of the difference of electro‐negativity of interlayer atoms, etc.	GPR	RMSE = 0.058 R^2^ = 0.949	When the layer number exceeds 8, the influence on the TMD bandgap decreases.	[54]
	C2DB, aNANt	2D materials	T_b_^mean^, C_g_^mean^, C_g_^std^, C_mol_^std^, H_e_^std^, T_m_^std^, r_cov_^std^, E_g_^PBE^	GPR	RMSE = 0.090 R^2^ = 0.990		[55]
Shear modulus	MD simulation	Graphene	Aspect ratio, temperature, vacancy defects, and number of atomic planes	GEP	RMSE = 0.004 R^2^ = 0.970	The number of defects has the most significant influence on the shear modulus.	[56]
Fracture mechanisms	MD simulation	Graphene	Fracture data	CNN		Increasing the flaw size to >3.2 nm will perturb the crack path.	[57]
	MD simulation	h‐WS_2_ and t‐WS_2_ monolayers	Chirality, system size, temperature, strain rate, random vacancy defect	RF	MSE = 9.2×10^−6^	Fracture strength decreases when temperature and the vacancy defect ratio are increased.	[58]
	MD simulation	MX_2_ (M = W, Mo and X = Se, S)	Varying crack lengths, chiral directions, simulation temperatures, and strains	LSTM and FFNN	LSTM MSE = 0.002 R^2^ = 0.998		[59]
Exfoliation energy	2DMatPedia	2D materials	Electrical properties, vdW interactions, etc.	MLR, ET, RT, and SVM	ET MSE = 0.005 MAE = 0.030 RMSE = 0.071 R^2^ = 0.890		[130]
Binding energy	DFT calculation	Each of the systems contained different nitrogen doped pores to stabilize single or pairs of metal atoms (Pt, Fe, Ni and Co)	Hundreds of general, molecular and statistical features	RF and SVM	RF MAE = 0.070 R^2^ = 0.990		[131]
	C2DB	2D materials	highest occupied molecular orbital, etc.	GBDT	MSE = 0.089 MAE = 0.214 RMSE = 0.289 R^2^ = 0.802	The energy levels of the highest occupied and lowest unoccupied molecular orbitals are crucial for binding energy.	[132]
Adsorption energy	DFT calculation	A single Li atom on regular MX_2_ and Janus MXY (M = W, Mo; XY = Te, Se, S) TMD structures	Electronic structure, atomic doping concentration	LR	MSE = 0.055 MAE = 0.044 R^2^ = 0.977	The side with higher electronegative chalcogen atoms is favorable for Li adsorption.	[133]
	C2DB, Materials Cloud, JARVIS‐DFT, 2DMatPedia	A single Li atom on 2D metallic materials	Work function of the 2D metal, ionization potential, coupling energy between Li^+^ and substrate	GCN and RF	GCN R^2^ = 0.770	Li adsorption energies can be affected by the 2D metal's work function and the Li^+^ and substrate coupling energy.	[134]
	DFT calculation	Alkali metal atoms on 2D materials	Element type and geometry descriptors	LR	MSE = 0.012 MAE = 0.080 R^2^ = 0.968	Alkali metal adsorption energies strongly correlate with the lowest unoccupied state energies of the materials.	[135]
	DFT calculation	MoS_2_, Au‐Cu clusters	Many‐body tensor representation or smooth overlap of atomic positions, atom‐centered symmetry functions	KRR	MAE = 0.100		[136]
Magnetic properties	C2DB	2D magnetic materials	Atomic and structural properties	RF	Accuracy = 92.5% F1 score = 0.860	3D transition metals, halides, and structural clusters with regular transition‐metal sublattices positively contribute to the magnetism.	[137]
	DFT calculation	Monolayers of the form A_2_B_2_X_6,_ based on the known material Cr_2_Ge_2_Te_6_	Atomic properties, formation energy	ETR, SVM, ANN, and KRR	ETR MAE = 0.330 R^2^ = 0.940	The X site significantly influences the magnetic coupling between neighboring A sites, driving the magnetic ordering.	[138]
	DFT calculation	Monolayers of the form A_2_B_2_X_6_, based on the known material Cr_2_Ge_2_Te_6_	Element properties	DT and ETR	ETR MAE = 0.46 R^2^ = 0.51	Hybridization between elements at the A and X sites in these systems is crucial for the magnetic moment.	[139]
*T_C_*	C2DB	2D magnetic materials	Crystal structure	GBR	MSE = 94.570	Both inter‐layer and intra‐layer exchange interactions are critical for determining *T_C_*.	[140]
	DFT calculation	2D magnetic materials	Spin hamiltonian parameters	DNN	MAE = 11.850 R^2^ = 0.994	The site's spin and exchange interactions proved to be the most significant factors affecting *Tc*.	[119]
Electrical breakdown limits	Self‐generated	Monolayer MoS_2_ devices with resistances and varying channel lengths	Current traces measured in the low‐voltage regime	DNN and CLSTM	CLSTM R^2^ = 0.830		[142]
Phononic properties	DFT calculation	Graphene, penta‐graphene, various haeckelite lattices, phagraphene and different graphyne structures	Moment tensor	MLIPs			[125]
	DFT calculation	Benzotrithiophene graphdiyne	Moment tensor	MLIPs			[143]
Electronic and structural properties	C2DB	VdW heterostructures	Monolayer properties of vdW heterostructures	EL	MAE = 0.034	VDW heterostructures exhibit differences in the equilibrium interlayer distance for AA and AB stacking configurations.	[144]
Mechanical properties	Materials Project, JARVIS‐DFT, C2DB	2D materials	Temperature, space group, vacuum size	XGBoost	MAE = 0.016 R^2^ = 0.880		[145]
Magnetic anisotropy energy	Self‐generated	2D metal−organic frameworks	Geometrical and elemental information	TMINN	RMSE = 0.011	In low‐coordinated or oxhydryl‐coordinated systems, 5d transition‐metal atoms are essential for magnetism.	[146]
Structural instabilities	C2DB	2D materials	Frequency of phonons	XGBoost	AUC = 0.910		[147]
Mechanical properties and thermal conductivity	DFT calculation	C_5_N	Moment tensor	MLIPs		Tensile strengths exceeding 10 Gpa and a low lattice thermal conductivity of ≈9.5 W m^−1^ K^−1^.	[126]
	DFT calculation	C_36_ fullerene network	Moment tensor	MLIPs		Room temperature phononic thermal conductivity and tensile strength are 9.8 ±1 W m^−1^ K^−1^ and 15.9 Gpa, respectively.	[148]
Diffusion Monte Carlo energies	DFT calculation	Graphene	Electron density, atomic environment vectors	VDNNs and KRR	KRR MAE = 3.400		[149]

### 3.1 Thermal Properties

#### 3.1.1 Thermodynamic Stability

**Figure 4:**
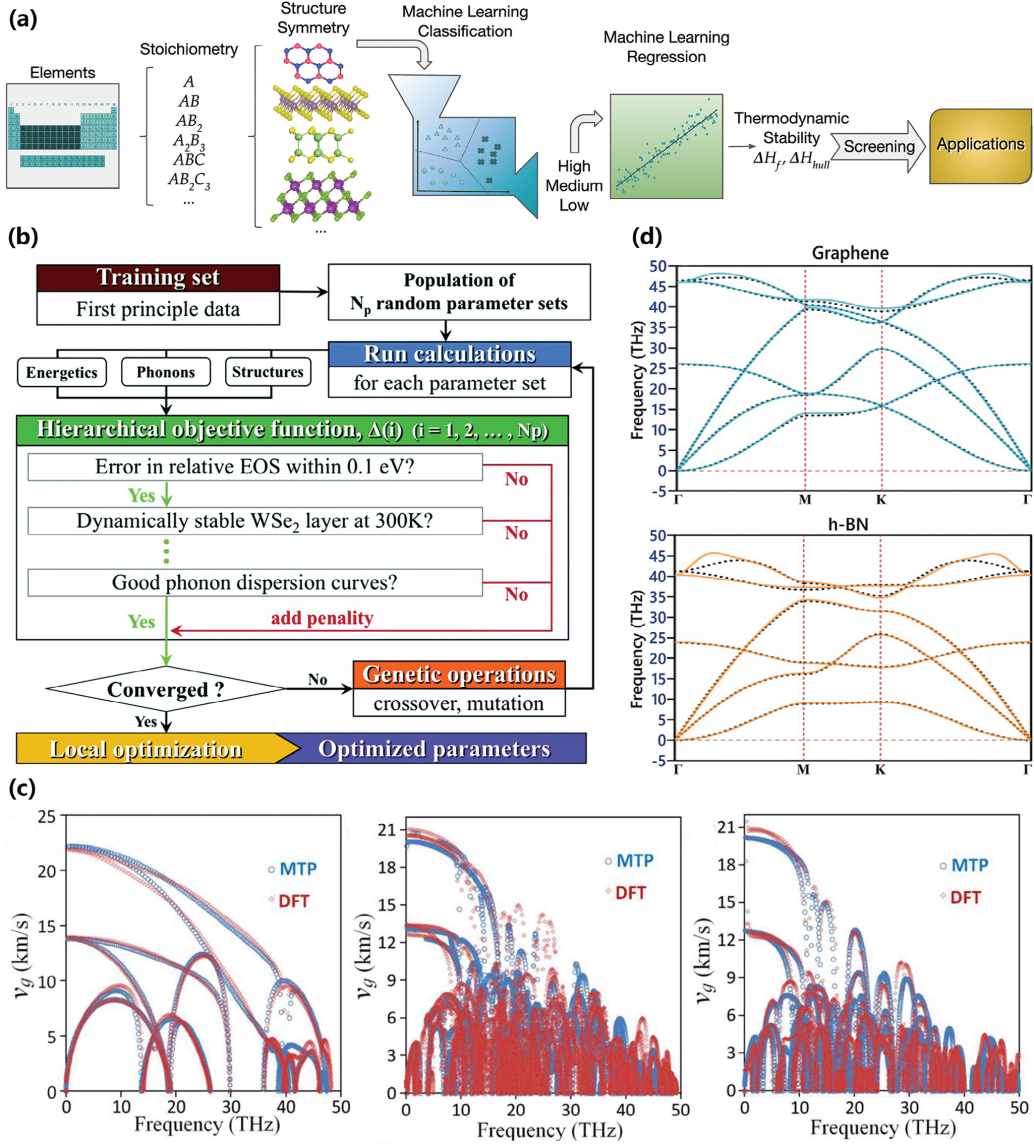
a) The process of thermal stability classification: the ML model descriptor is comprised of basic information such as the element and atomic structure, and the 2D materials to be predicted are partitioned into stable and unstable materials according to their *ΔH_f_* and *ΔH_hull_*_._ Reproduced with permission.^[^ ^17^ ^]^ Copyright 2020, American Chemical Society. b) The ML training workflow for WSe_2_ interatomic potentials. The training data comes from DFT simulations, and a hierarchical objective function is used to assign weights to the features in a non‐random way. Global and local optimization are used to find the best parameters. Reproduced with permission.^[^ ^33^ ^]^ Copyright 2019, Royal Society of Chemistry. c) A trained MTP model can closely reproduce the phonon group velocity of monolayer phagraphene, graphene, and haeckelite, which can displace DFT calculations to obtain the anharmonic atomic force constant. Reproduced with permission.^[^ ^36^ ^]^ Copyright 2021, Elsevier. d) The phonon dispersion relations of graphene and h‐BN obtained by the MLIPs model (dashed line) and by DFT calculations (solid line) are consistent, and the MLIPs model can accurately predict the thermal expansion coefficient. Reproduced with permission.^[^ ^44^ ^]^ Copyright 2019, American Physical Society.

Thermal stability is a fundamental factor to consider in high‐throughput screening of 2D materials. In the study conducted by Schleder et al, the thermodynamic stability of non‐magnetic materials was analyzed based on the C2DB database.^[^ ^17^ ^]^ Specifically, they used the structural and atomic properties of the materials as the feature set and applied the SISSO method to construct a feature space and obtain the descriptors. These descriptors were derived from nonlinear combinations of different features and used to classify materials with a given structure into stable and unstable materials based on their energy above the convex hull (*ΔH_hull_*) and formation energy (*ΔH_f_*), as shown in **Figure** **4**a. They also employed a stochastic gradient boosting decision‐tree classifier to evaluate the contribution of each descriptor to the output predictions. Finally, they selected the six best descriptors and incorporated them into the classifier. The study demonstrated the importance of periodic group and the electron affinity in describing thermal stability. Although the researchers did not explore the thermal stability of more complex magnetic materials, their method successfully predicted thermal stability by relying solely on the prototype structure in the absence of exact information about the atom site.

#### 3.1.2 Thermal Conductivity

Thermal conductivity describes the ability of a material to transfer heat, which is the sum of phononic and electronic contributions. Hence, for predicting thermal conductivity through a ML model, it is crucial to incorporate phonons as features in the model training process, even though phonon calculations by themselves are computationally costly. Furthermore, when calculating thermal conductivity using classical MD simulations, interatomic potential also plays a crucial role. Therefore, many research works have focused on using ML algorithms for interatomic potentials.^[^ ^33^ ^]^ The 2D semiconductor, WSe_2_, has an extremely low thermal conductivity that is comparable to that of electric‐insulation glass. Chan et al. developed ML‐BOP, a bond‐order‐potential model of the W‐Se system based on supervised ML that combines global and local optimization.^[^ ^33^ ^]^ The model uses their self‐designed Tersoff‐Brenner function to describe the interatomic potential of WSe_2_, and the captured phonon dispersion relation and density of state are consistent with the results of DFT calculations. This means that their model has the capability to make precise predictions for the thermal conductivity of monolayer, multi‐layer, disordered, and other low‐dimensional nanostructures of WSe_2_. The research uses the least‐squares method and linear regression (LR) to minimize the feature data, which includes lattice parameters, elastic properties, equations of state, cohesive energies, and phonon dispersion, obtained by DFT calculations. The GA and Nelder‐Mead Simplex algorithm are then employed to generate or select candidate features. Among them, the GA is for the global optimization of layered objects, while Nelder‐Mead Simplex is for searching the local optimum. Combining the two can generate a set of optimal BOP parameters of WSe_2_ (Figure 4b). Mortazavi et al. used the momentum tensor potential (MTP) as an accurate and efficient model for describing interatomic forces.^[^ ^36^ ^]^ Based on the interatomic potentials of some 2D materials, MLIPs trained over short *ab‐initio* molecular dynamics (AIMD) trajectories could replace DFT calculations to obtain the anharmonic atomic force constant. The researchers used popular non‐equilibrium MD simulations with a fitted MLIPs to estimate the thermal conductivity of polyaniline C_3_N monolayer. Compared with the first‐principles calculation based on DFT plus the Boltzmann transport equation, their method not only successfully reproduced the phonon group velocities and phonon dispersions but also improved the accuracy of classical MD simulations (Figure 4c).

#### 3.1.3 Thermal Expansion

Materials are known to expand as their temperature increases, but predicting the thermal expansion of 2D nanomaterials remains a challenge. While AIMD simulations can provide very precise estimates, their computing costs are high. Conversely, classic MD simulations come with more affordable computing costs, but cannot achieve the required level of accuracy. To address this, Mortazavi et al. employed MD simulations to calculate the linear thermal expansion coefficient (TEC) of carbon‐based nanosheets, in which the difference in TEC between structures can be attributed to the atom type, atomic configuration, wrinkle amplitude, bond strength, and density.^[^ ^44^ ^]^ Not only could their trained MLIPs enable the investigation of thermal expansion in complex nanomembranes across a broad temperature range, but it could also exhibit excellent accuracy in reproducing the phonon dispersion relations and TEC predicted by DFT calculations (Figure 4d). In addition, based on the MLIPs algorithm, Ali Rajabpour et al. also investigated the effect of a substrate on the TEC of various 2D materials, including C_3_B, C_3_N, graphene, and phagraphene monolayers.^[^ ^45^ ^]^

### 3.2 Electrical Properties

#### 3.2.1 Electronic Band Structure

**Figure 5:**
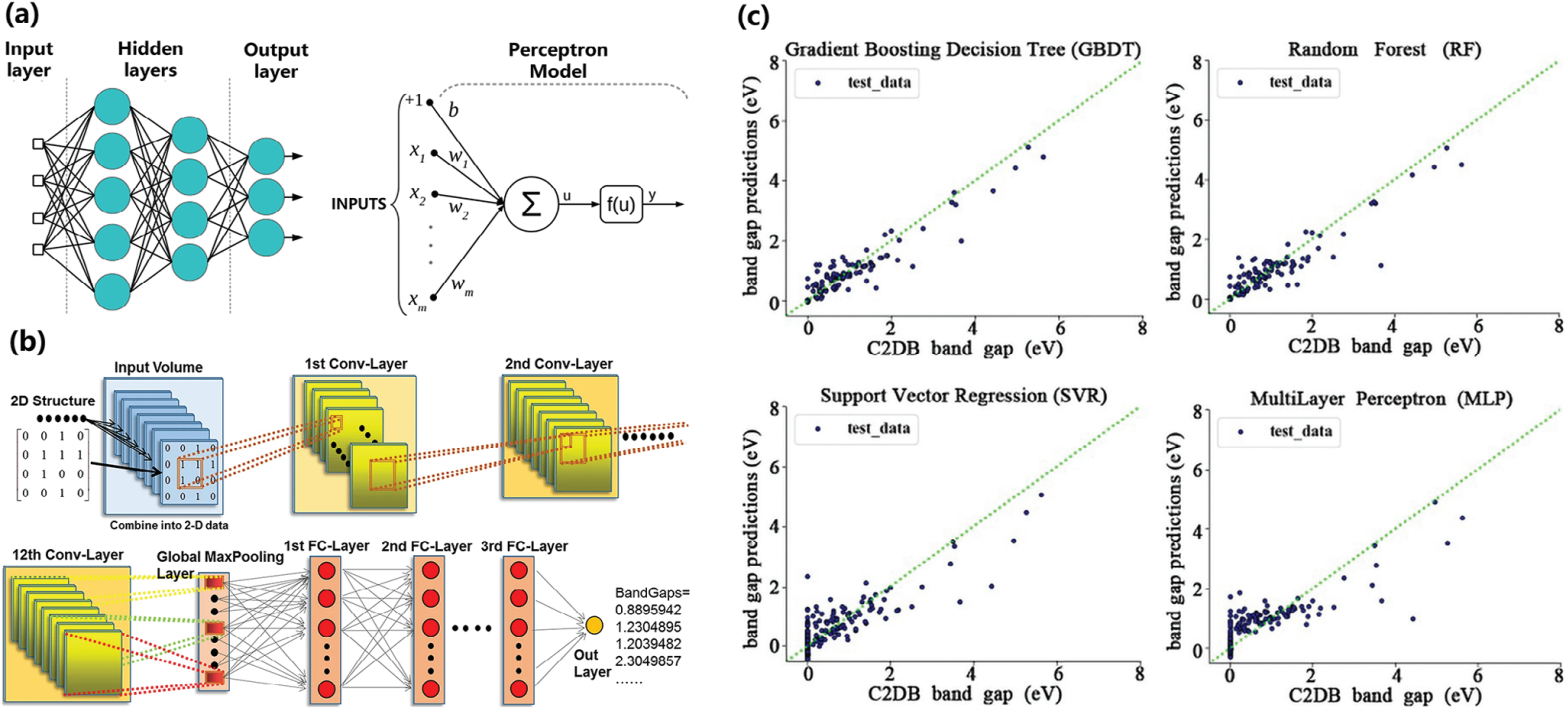
a) An ML model used for predicting energy band structures of 2D semiconductors, that consists of an ANN with two hidden layers (left) and a perceptron (right).^[^ ^129^ ^]^ b) The CVN architecture for predicting the bandgap of 2D hybridized graphene, consisting of 12 convolution layers, one global max‐pooling layer, three fully‐connected layers, and an output layer. Reproduced with permission.^[^ ^47^ ^]^ Copyright 2019, Springer Nature. c) The fitness of predicted bandgap to actual value was evaluated by comparing ML predicted value with C2DB calculated value. RF and GBDT results showed strong linear correlations with the calculated values, while SVR and MLP results had weaker correlations. Reproduced with permission.^[^ ^48^ ^]^ Copyright 2021, Public Library of Science.

The electronic band structure is a fundamental feature of solid crystals. Thygesen et al. have constructed the features based on radially decomposed projected density of states and energy decomposed operator matrix elements, and have used the output of DFT calculations on the dataset of non‐magnetic 2D semiconductors as inputs to a gradient boosting (GB) model to predict the complete energy band structure of G_0_W_0_.^[^ ^127^ ^]^ In addition, by applying the resulting ML model, they predicted G_0_W_0_ band structures for ≈700 2D semiconductors from the C2DB. Ferreira et al. constructed multilayer perceptron and ANN (**Figure** **5**a) based on 2D and 3D photonic crystals made of different lattices, geometries, and materials, which could quickly compute the photonic bandgaps and energy band structure of 3D and 2D photonic crystals.^[^ ^129^ ^]^

#### 3.2.2 Bandgaps

There have been many publications on the bandgap properties of 2D materials. Dong et al. proposed a material descriptor for hybridized boron‐nitrogen graphene with various supercell configurations.^[^ ^47^ ^]^ This descriptor enables the identification of correlations between the structure and bandgap, where localized atomic clusters collectively determine the bandgap of the entire structure through the interactions between neighboring atoms. They further trained CNN models, including residual convolutional networks (RCN), VGG16 convolutional networks (VCN), and concatenate convolutional networks (CCN) using this descriptor. This model successfully predicted the bandgaps of hybridized graphene and boron nitride pairs with arbitrary configurations, achieving an accuracy above 90%. Figure 5b shows this VCN architecture. Zhang et al. predicted the bandgap values of 2D materials based on C2DB by four algorithms, namely, support vector regression (SVR), multilayer perceptron (MLP), gradient boosting decision tree (GBDT), and random forest (RF).^[^ ^48^ ^]^ Through various experiments, they discovered that GBDT and RF performed better in predicting bandgap values of 2D materials than did the other two algorithms (Figure 5c), where three features, namely, density of states at the Fermi energy, heat of formation, and gap without spin‐orbit coupling, had a great impact on the model performance. Rajan et al. constructed a database of the structural and electrical properties of 7200 MXenes and then reduced the number of features to eight by using LASSO.^[^ ^10^ ^]^ The Gaussian process regression (GPR) model trained on these features could accurately estimate the bandgaps of the entire MXenes database in just a few minutes. Wang et al. studied the electrical properties of vdW heterostructures of layered 2D transitional metal dichalcogenides (TMDs) using five ML models, and found that the electrical properties were greatly influenced by the layer number of 2D material.^[^ ^54^ ^]^ They also discovered that the GPR model performed better in predicting bandgaps than the other four models.

### 3.3 Mechanical Properties

#### 3.3.1 Shear Modulus

**Figure 6:**
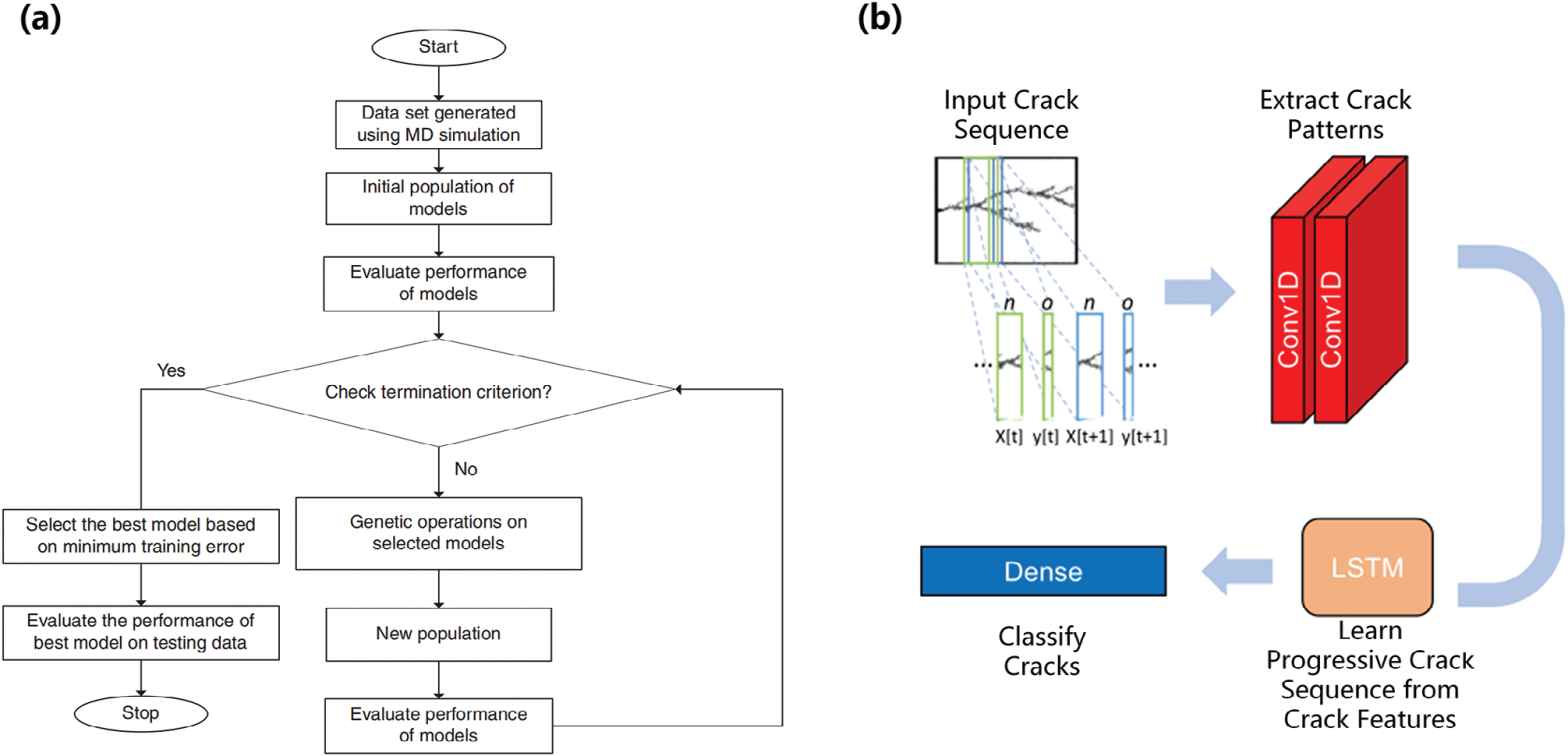
a) The ML model, trained on MD simulations, utilizes the GEP algorithm to predict the shear modulus of graphene. The GEP algorithm, similar to the GA, employs tree structures of different lengths to search for the optimal structure. Reproduced with permission.^[^ ^56^ ^]^ Copyright 2015, American Physical Society. b) An ML model for predicting fracture toughness of graphene, which consists of two convolution layers that learn geometric features of crack slices, a long short‐term memory (LSTM) layer that learns sequential relations between them, and a dense layer that classifies the results. Reproduced with permission.^[^ ^57^ ^]^ Copyright 2021, Springer Nature.

Graphene, because of its unique mechanical properties, has been an ideal candidate for the application of fluid separation, nanofiltration and nanoelectromechanical systems. Garg et al. proposed an ML model (**Figure** **6**a) trained on MD simulations that could establish an explicit relationship between the shear modulus of graphene nanostructures and various system parameters, such as temperature, vacancy defects, number of atomic planes, and aspect ratio.^[^ ^56^ ^]^ The shear modulus predicted by their MD‐based ML model was consistent with the results of existing experiments, and they found that the shear modulus of graphene nanostructures is primarily affected by the quantity of defects.

#### 3.3.2 Fracture Toughness

Understanding fracture toughness is crucial when it comes to the design of elastic nanomaterials. Lew et al. utilized a convolutional long short‐term memory model (CLSTM) (Figure 6b) to predict the fracture mechanism of graphene by extracting spatiotemporal relationships underlying fracture propagation from MD simulations datasets.^[^ ^57^ ^]^ Their ML approach enabled rapid prediction of crack instabilities and branching behaviors, thereby enhancing the capacity to design and optimize fracture behaviors according to specific requirements. In another study, Wang et al. evaluated the mechanical properties, including Young's modulus, fracture strength, and fracture strain, of 1T‐WS_2_ and 2H‐WS_2_ monolayers by MD simulations and an ML technique based on five features: the WS_2_ phase, temperature, strain rate, chirality, and defect ratio.^[^ ^58^ ^]^ They found that the RMSE were significantly smaller than the actual values of each property, indicating a well‐trained ML model with good prediction accuracy for the mechanical properties.

### 3.4 Energy

#### 3.4.1 Exfoliation Energy

**Figure 7:**
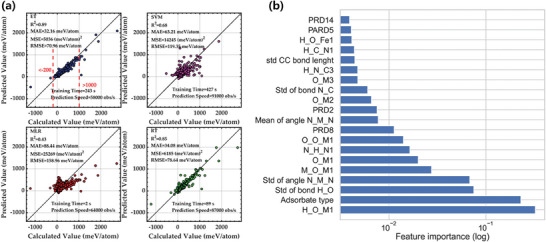
a) Scatter diagrams of exfoliation energy predictions obtained by different ML models. The fitting degree shows that ET achieves the highest prediction accuracy, followed by RT. Reproduced with permission.^[^ ^130^ ^]^ Copyright 2021, American Chemical Society. b) The top 20 most important features that affect the bond energy of porous graphene‐based monatomic metal catalysts. Reproduced with permission.^[^ ^131^ ^]^ Copyright 2020, John Wiley and Sons.

The exfoliation energy is a direct indicator of the ease with which monolayers can be mechanically exfoliated from bulk compounds. Wan et al. manually selected 12 descriptors for predicting exfoliation energy from the 2DMatPedia database (including six related to vdW interactions, five related to electrical properties, and one decomposition energy that describes the stability of bulk materials) to train four ML models—SVM, multilinear regression (MLR), ensemble trees (ET), and regression tree (RT) models.^[^ ^130^ ^]^ The performance of these models was assessed by MAE, RMSE and coefficient of determination. Extensive validations and stability analysis show that ET and RT algorithms can process features better and hence perform better at making predictions (**Figure** **7**a).

#### 3.4.2 Binding Energy

The binding energy of small molecules is a key factor in dictating the reaction efficiency and application range of single‐atom catalysts. To predict the binding energy of single metal atoms to N‐doped graphene defects, Fischer et al. employed a random forest regression (RFR) model trained on ≈1700 catalytic reaction simulations generated by DFT calculations.^[^ ^131^ ^]^ Their study involved constructing three distinct structural feature groups, including hundreds of chemical features, statistical features, and molecular features, as well as employing a correlation matrix to identify and remove strongly correlated features to improve model performance. Figure 7b illustrates the logarithmic scale of the 20 essential bond energy features, with half of them being related to bond angles. The RFR approach proved to be suitable for handling small datasets with a vast feature space, achieving an accuracy of 0.865 in predicting bond energy.

#### 3.4.3 Adsorption Energy

Lithium (Li) adsorption is a crucial electrochemical process in material applications. Gong et al. compiled data from five databases (C2DB, Materials Cloud, Jarvis, 2DMatPedia, and Jain) into a comprehensive database with 7736 2D materials, and used a graph convolution network (GCN) to predict the minimum Li adsorption energy of 2D metallic materials.^[^ ^134^ ^]^ The GCN was trained using DFT‐calculated Li site energies of various adsorption sites for each material, and was able to identify a strong correlation between the minimum Li adsorption energies and the coupling energy between Li+ and the substrate, the work function of the 2D metal, and the sum of ionization potential. Notably, for zero‐gap 2D materials, the minimum Li adsorption energy was found to strongly correlate with the position of the lowest unoccupied band or work function. In addition, Dou et al. found that simple regression algorithms can effectively predict the adsorption energy of alkali metal atoms on different monolayer 2D TMDs using several groups of descriptors—the ionization energy of adsorbates, the cohesive energy of adsorbate crystals, and the lowest unoccupied states of 2D TMDs.^[^ ^135^ ^]^

### 3.5 Other Properties

#### 3.5.1 Magnetic Properties

**Figure 8:**
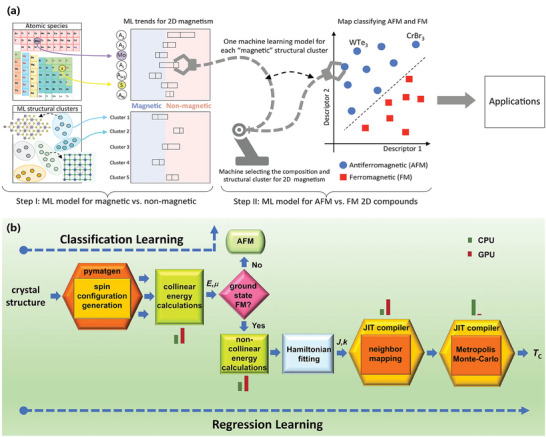
a) The RF model used to identify and predict the existence of magnetism in the given 2D compounds (Step I) and their specific magnetic orders (Step II). Reproduced with permission.^[^ ^137^ ^]^ Copyright 2022, American Chemical Society. b) The ML model for predicting *T_C_* of 2D materials, which classifies the materials into ferromagnetic and antiferromagnetic based on symmetry, and then the *T_C_* of the FM materials is estimated by using Heisenberg model‐based Monte Carlo simulations. Reproduced with permission.^[^ ^140^ ^]^ Copyright 2020, Springer Nature.

Magnetic 2D materials have been recognized as fundamental building blocks for spintronic applications, which are crucial in extending data storage and quantum device technologies.^[^ ^150^, ^151^, ^152^ ^]^ However, the efficient search of such 2D materials and corresponding experimental realization remain quite challenging, due to various complications that can occur in practice.^[^ ^153^, ^154^, ^155^, ^156^ ^]^ To this end, based on magnetic and non‐magnetic compounds in the C2DB database, Acosta et al. utilized the RF algorithm and the Shapley additive explanations method to predict the magnetic ordering of 2D materials (**Figure** **8**a).^[^ ^137^ ^]^ They first constructed a feature space by SISSO and then employed a RF model to identify features for the classification of materials. According to their study, the presence of halides, 3D transition metals, and structural clusters with regular transition‐metal sublattices positively contributes to the total weight that determines magnetism in 2D compounds. This behavior is attributed to the competition between crystal field and exchange splitting. The study also found that atomic spin‐orbit coupling is a key feature for distinguishing between ferro‐ and antiferromagnetic orders. In Rhone et al.’s work, they explored the magnetic order and magnetic moment of monolayer A_2_B_2_X_6_ by DFT calculations and ML based on the known ferromagnetic semiconductor Cr_2_Ge_2_Te_6_.^[^ ^138^ ^]^ More specifically, the atomic attributes from python mendeleev package 0.4.1 were used as descriptors for their ML model, an extra tree regression (ETR) algorithm was used to predict magnetic moments, and then the SVM classifier was employed for predicting low‐energy magnetic orders. Their experiments demonstrated that the magnetic coupling near A sites is strongly influenced by the X site and can trigger magnetic ordering. As a result, the magnetism of monolayer A_2_B_2_X_6_ can be regulated by atomic exchange between the A, B and X sites.

#### 3.5.2 Curie Temperature

The *T_C_* is a critical parameter for 2D ferromagnetic materials. Based on the C2DB database and on first‐principles calculations, Kabiraj et al. developed a computer code that could perform Heisenberg model‐based Monte Carlo simulations to predict the *T_C_* from crystal structures, as shown in Figure 8b.^[^ ^140^ ^]^ Their method successfully computed the *T_C_* of 157 materials, of which 26 had a *T_C_* above 400 K. The ML model they constructed was trained on the data of these 157 materials to successfully identify 2D ferromagnetic materials with a higher *T_C_* than those in other databases.

#### 3.5.3 Electrical Breakdown Limits

Identifying the limits of electrical breakdown for materials is a crucial step in the development of electronic devices. Using the electric current measured with the low‐voltage area as inputs, Huan et al. trained a two‐step DL model to investigate the breakdown voltage of single‐layer MoS_2_ devices with different resistances and channel lengths.^[^ ^142^ ^]^ In their two‐step model, a DNN first classifies between avalanche and joule breakdown mechanisms by analyzing partial current traces ranging from 0 to 20 V. After that, a CLSTM, which combines the complementary methods of CNN and LSTM, estimates breakdown voltages of the devices. Experiments show that DNN classifier achieved a high accuracy of 79% in classifying the breakdown mechanisms, while the CLSTM model had a low error rate of only 12% in predicting the breakdown voltages. This method is expected to facilitate prompt and non‐destructive material characterization for the development of 2D electronic devices.

## 4 Discovering New 2D Materials

The introduction of ML to research on 2D materials has considerably increased the efficiency of discovering new 2D materials. As shown in **Table** **3**, most of these new materials are catalytic materials,^[^ ^60^, ^61^, ^62^, ^63^, ^64^, ^65^, ^66^, ^67^, ^68^ ^]^ photoelectric materials,^[^ ^69^, ^70^, ^71^, ^72^, ^73^, ^74^, ^75^, ^76^, ^77^ ^]^ and ferromagnetic materials.^[^ ^78^, ^79^, ^80^, ^81^, ^82^, ^83^, ^84^, ^85^, ^90^, ^91^, ^92^ ^]^ In most cases, we search for 2D materials with specific desired properties in existing open‐source databases or in new material sets generated through methods such as element replacement within the original cell. In the study by Lyngby et al., the collection of 2D materials from the C2DB was served as seed structures for the lattice decoration protocol and employed as the training dataset for the crystal diffusion variational autoencoder.^[^ ^30^ ^]^ The former generates new materials by substituting the atoms in the seed structures with atoms of similar chemical nature, while the latter combines a variational autoencoder and a diffusion model to generate new periodic materials, capable of producing more complex materials without compromising stability. After performing DFT relaxation on the structures generated by these two methods, any duplicate structures and materials that relaxed into non‐2D structures were subsequently discarded, resulting in a total of 11630 predicted new 2D materials. In the development of new 2D materials, classification algorithms such as k‐nearest neighbor (KNN), SVM, RF, ANN, and GB are commonly employed to screen materials with specific desired properties, with GB being the most extensively utilized.

**Table 3:** Summary of the ML‐enabled development of new 2D materials. (Full names of the ML algorithms are listed in Appendix).

Research area	Data source	ML algorithms	Assessment metrics	Targets	Achievements	Ref.
NRR electrocatalysts	CSD	LGB		Predicting efficient nitrogen reduction reaction (NRR) electrocatalysts	9 efficient NRR electrocatalysts	[60]
Catalysts for the hydrogen evolution reaction (HER)	The database of 420 types of stable O atom terminated 2D Mxenes OBAs	SVR, GPR, RFR, and Adaboost	Adaboost RMSE = 0.140 R^2^ = 0.950	Identifying 2D Mxenes ordered binary alloy (OBA) activity trends and guiding hydrogen evolution reaction (HER) catalyst design	110 ordered binary alloys of Mxenes for HER catalysts	[61]
	Self‐generated	GBR	AUC = 0.941	Understanding the HER activity of doped single‐atom catalysts on 2D GaPS_4_	44 stable anchoring materials for HER catalysts	[62]
	Self‐generated	LGB	MAE = 0.224 R^2^ = 0.951	Searching for 2D lateral heterostructures with HER	7 lateral heterostructures for HER catalysts	[63]
High‐performance electrocatalysts	Self‐generated	RF and ANN	ANN R^2^ = 0.740	Screening 2D‐TMDs with high HER activity	9 TMDs with high HER activity	[64]
Octahedral 2D photocatalysts	aNANt	RFE,LGB, XGBoost, LR, RF, and LASSO	LGB MAE = 0.099 RMSE = 0.131 R^2^ = 0.979	Identifying stable octahedral 2D photocatalysts for water splitting	21 potential stable octahedral 2D photocatalysts	[65]
2D multinary component photocatalysts	C2DB	GBR, KNR, ABR, LR, and RFR	GBR RMSE = 0.340 R^2^ = 0.943	Selecting eligible candidates for photocatalytic water splitting	75 multinary compounds for photocatalytic water splitting	[66]
Superb hydrogen evolution electrocatalysts	Self‐generated	ERT	RMSE = 0.180 R^2^ = 0.880	Screening transition metal single atom based superb hydrogen evolution electrocatalysts	20 stable superb hydrogen evolution electrocatalysts	[67]
2D Mxenes HER electrocatalysts	Self‐generated	KNN	RMSE = 0.110 R^2^ = 0.860	Applying a ML model to capture the trend in catalytic activity	5 single transition metal doped Ti_2_CO_2_ materials with high HER activity	[68]
2D Mxenes	C2DB	CGCNN	MAE = 0.085 RMSE = 0.093	Predicting the heat of formations, bandgaps, and in‐plane elastic tensor components of 2D Mxenes		[69]
Photoelectric (PE) materials	Aflow, Materials Project, OQMD, COD	DT, KNN, MLP, GBDT, and RF	LGB AUC = 0.900	Identifying potential solar absorber materials	58 potential 2D thin‐film solar absorber materials	[70]
2D photoelectric (2DPE) materials	ICSD	SGDC, SVM, Adaboost, LR, GB, RF, and DT	GB AUC ≈ 1	Identifying the 2DPE candidates from a database of known materials with high efficiency and accuracy	26 2DPE materials	[71]
	Self‐generated	RF and GA	RF MSE = 0.203 R^2^ = 0.994	Searching for 2D perovskite materials suitable for photoelectric applications	3 possible perovskite materials	[72]
	PubChem database	EL	MAE = 0.484	Searching for interface passivation materials for perovskite solar Cells	10 ammonium salts with high ratios of power conversion efficiency	[73]
2D materials for energy conversion and storage	C2DB	ANN	MAE = 0.064 R^2^ = 0.980	Accelerating the virtual screening of 2D materials and the discovery of new candidates with targeted physical and chemical properties		[74]
2D germanene‐based Janus materials	Self‐generated	KRR, ETR, ABR, RF, and SVR	SVR MAE = 0.032 RMSE = 0.042 R^2^ = 0.971	Designing novel Janus 2D germanene‐based materials for photoelectric or photocatalytic applications	23 Ge_8_H_n_X_8‐n_ structures with photovoltaic or photocatalysis potential	[75]
2D halide perovskites	2D ion/perovskite systems database	RF, SVM, XGBoost, and KNN	XGBoost RMSE = 0.760 R^2^ = 0.940	Evaluating the interactions between the ions and the 2D halide perovskites toward the energy storage applications	5 potential candidates for ion capacitors	[76]
	Self‐generated	SVR and XGBoost	XGBoost MAE = 0.070 R^2^ = 0.970 RMSE = 0.100	Searching 2D perovskite materials with tailored bandgaps	18 organic–inorganic halide perovskites with tailored bandgaps	[77]
Ferromagnetic semiconductors	2D M_I_M_II_Ge_2_X_6_ family	GB		Finding 2D ferroic materials and room‐temperature FM semiconductors	12 stable multiferroics and 35 stable room‐temperature FM semiconductors	[78]
2D ferroelectric metals	2D M_I_M_II_Ge_2_X_6_ family	SVC, RF, DT, Adaboost, and GB	GB Accuracy = 77.2% AUC = 0.883	Accelerating the discovery of ferroelectrics from 2964 structures of M_I_M_II_Ge_2_X_6_ materials	60 novel stable ferroelectric materials	[79]
2D ferromagnets	C2DB	XGBoost	MAE = 0.030 R^2^ = 0.989	Identifying promising 2D ferromagnets with high *T_C_*	Over 700 potential FM materials with high *T_c_*	[80]
	C2DB	GB	AUC = 0.963	Discovering stable 2D FM semiconductors, half‐metals, and metals	20 FM semiconductors, 21 FM half‐metals, and 51 FM metals	[81]
	Self‐generated	RFR	R^2^ = 0.940	Screening 2D ferromagnetic materials	1225 ferromagnetic materials	[82]
	Self‐generated	RF	F1 score = 0.640 AUC = 0.650	Discovering novel 2D TMDs with large magnetic anisotropies	11 structures with an anisotropy greaterthan 1 meV	[83]
Magnetic topological insulators	Self‐generated	CNN	Accuracy = 94.8%	Discovering 2D magnetic topological insulators	5 magnetic topological insulators	[84]
	Self‐generated	RFR, ETR	ETR MAE = 0.200 R^2^ = 0.940	Discovering novel topological magnetic vdW magnets in the MnBi_2_Te_4_ family	13 materials in the MnBi_2_Te_4_ family	[85]
2D magnets	Self‐generated	GNBC		Exploring 2D magnetic materials	8 stable magnetic materials	[9]
	Self‐generated	NN	R^2^ = 0.800	Exploring 2D vdW magnets with chemical stability	3 stable vdW magnets	[90]
	C2DB, Materials Cloud	SVM, RF, and KRR	RF MAE = 0.150 R^2^ = 0.810	Screening candidate materials for 2D magnets with high magnetization	7 stable magnetic materials with high anisotropy energy	[91]
	2DmatPedia, C2DB and Materials Cloud	EA		Looking for materials with hole doping to induce ferromagnetism	122 materials exhibiting a hole‐doping induced ferromagnetism	[92]
vdW materials	Materials Project	RF	Accuracy = 78.2%	Developing a framework for discriminating between vdW and non‐vdW materials		[157]
	Materials Project, ICSD	SVM	P = 100.0% R = 81.0%	Discovering materials likely to exhibit layered and 2D phases	10 stable layered materials.	[86]
	Materials Project, ICSD	ML		Exploring 2D layered materials and materials composed of weakly bonded 1D molecular chains	1173 layered materials and 487 materials that consist of weakly bonded 1D molecular chains	[87]
2D vdW heterostructures	JARVIS‐DFT	ALGNN	MAE = 0.230	Accelerating the design and discovery of 2D vdW heterostructures	75745 type‐I, 85722 type‐II, and 65312 type‐III 2D heterostructures	[158]
	Self‐generated	XGBoost and LR	XGBoost AUC = 0.890	Finding high‐performance 2D M−S vdW heterostructures with low resistance for potential applications to electronic devices	6 novel vdW heterostructures	[159]
Janus 2D III−VI vdW heterostructures	The Janus III−VI vdW heterostructure database	CGCNN	MAE = 0.001 RMSE = 0.002 R^2^ = 0.999	Accelerating the discovery of promising photocatalytic and photoelectric candidates in Janus III−VI vdW heterostructures	1035 Janus III‐VI vdW heterostructures	[160]
2D doped tellurene for fin field‐effect transistor devices	Self‐generated	XGBoost	AUC = 0.800	Discovering proper 2D doped tellurene for fin field‐effect transistor devices	23 proper materials for fin field‐effect transistor devices	[89]
2D TMDs materials for Hg^0^ sensory application	Aflow	ML		Accelerating the discovery of 2D TMDCs materials for Hg^0^ sensory application	6 TMDCs materials for the detection of Hg°	[161]
2D transition metal carbides, carbonitrides, and nitrides	Mxenes	PUML		Quantifying the degree of “synthesizability” of theoretically predicted MAX and Mxenes compounds	111 MAX phases and 18 Mxenes can be synthesizable	[162]
Charge density wave (CDW) materials	Self‐generated	MS		Finding the Charge density wave (CDW) phases of any 2D material	35 nonmagnetic materials with potential nontrivial CDW behavior	[163]
New hypothetical 2D materials	2D Materials, Materials Project, ICSD	RF	F1 score = 0.890 AUC = 0.960	Discovering novel 2D materials in uncharted composition space	101 crystal structures and 92 layered materials	[164]
2D transition metal dichlorides	2DmatPedia	GGA		Screening out a family of transition metal dichlorides	6 stable transition metal dichlorides	[165]
Novel solid lubricant and super‐lubricant materials	2DmatPedia	BNN	MAE = 0.010 RMSE = 0.014 R^2^ = 0.980	Creating a dataset of structural properties of 18 million vdW layered structures	60910 stable structures at room temperature	[166]
New 2D structures	C2DB	ANN		Finding new 2D structures, either in vacuum or on a substrate		[167]
New 2D materials	C2DB	DL, RF	RF MAE ≈ 13.550 RMSE ≈ 24.050 R^2^ ≈ 0.890	Searching for structurally stable 2D materials	760 potential stable materials	[168]
3D and 2D thermoelectric materials	JARVIS‐DFT	DT, RF, KNN, MLP, and GBDT	LGB AUC = 0.960	Identifying efficient thermoelectric materials	2932 3D and 148 2D efficient thermoelectric materials	[169]
2D topological insulators	C2DB, 2DmatPedia	XGBoost	F1 score = 0.800 AUC = 0.960	Searching the materials space for 2D topological materials	56 topological materials	[170]
2D materials for water desalination	C2DB	LR, KNN, RF, and XGBoost	XGBoost MAE = 0.010 R^2^ = 0.990	Predicting the desalination performance of 2D membranes that exist in the literature	3814 materials for water desalination	[171]
2D materials with flat electronic bands	2DmatPedia	ANN and UMLBC		Finding 2D materials with flat bands		[172]
2D nonlinear optical materials	Self‐generated	ML		Predicting 2D nonlinear optical materials	46 nonlinear optical materials	[173]

### 4.1 Boosting Algorithm

Boosting is an ensemble learning technique that combines multiple learners to finish a learning task.^[^ ^174^ ^]^ Based on the generation method of individual learners, the ensemble learning methods can be broadly divided into two categories.^[^ ^175^ ^]^ the first category includes methods represented by boosting, which have a strong correlation between individual learners and a serialization method that must be generated sequentially, while the second category includes methods in which there is no dependency between individual learners and a parallelization method, which can be generated at the same time, such as the RF algorithm.^[^ ^176^ ^]^ In the case of boosting, a base learner is first trained using the initial training set. Then, the distribution of the training samples is adjusted based on the performance of the base learner, and a new base learner is trained using the adjusted sample distribution. This process is repeated to train the specified number of learners, and a strong learner is formed by combining all base learners using their trained weights.^[^ ^177^ ^]^

Classical boosting algorithms include GBDT, extreme gradient boosting (XGBoost), adaptive boosting (Adaboost), light gradient boosting (LGB), and categorical boosting.^[^ ^178^ ^]^ GBDT is an iterative decision tree algorithm, which constructs multiple decision trees and summarizes the outputs of all the trees to generate the final result for classification.^[^ ^179^ ^]^ GBDT can flexibly process data of different types, boasts a strong level of robustness in dealing with anomalies, and achieves higher levels of accuracy than SVM under the same fine‐tuning of parameters.^[^ ^180^ ^]^ For instance, Choudhary et al. used ML to identify promising solar cell materials from the publicly available JARVIS‐DFT database.^[^ ^70^ ^]^ The ML models are trained using decision‐trees (DT), RF, KNN, MLP, and GB models implemented in the scikit‐learn package, and also GBDT implemented in the XGBoost and LightGBM packages. The accuracies of the classification models were evaluated based on the area under the curve (AUC) of the receiver operating characteristics (ROCs) curves, illustrating the model's ability to identify potential solar absorber materials.^[^ ^70^ ^]^ The higher the value of the AUC, the higher the accuracy of the corresponding model. Among the above several algorithms, the LGB algorithm achieved the highest AUC value of 0.87 and was therefore considered the best model.

**Figure 9:**
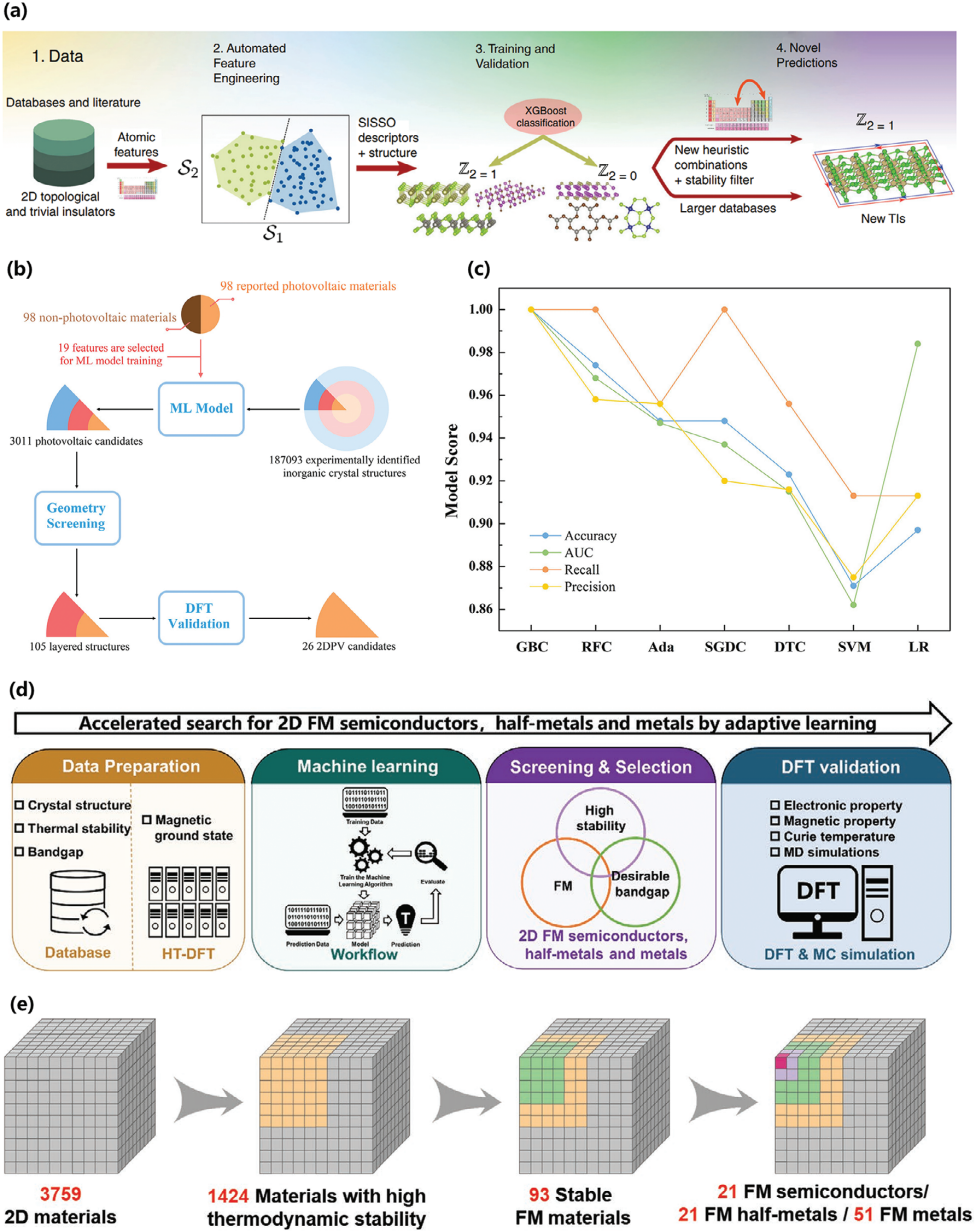
a) Discovery workflow of 2D topological materials: first, an initial dataset is built. Next, SISSO is employed to convert atomic features into topological classes; the XGBoost method is employed to train the ML model. Finally, the ML model searches for novel topological materials. Reproduced with permission.^[^ ^170^ ^]^ Copyright 2021, American Institute of Physics. b) The screening process of 2DPE materials. Reproduced with permission.^[^ ^71^ ^]^ Copyright 2021, American Chemical Society. c) Following (b), a comparison of the performance of different algorithms by four measures—accuracy, AUC, recall and precision, with the GB algorithm being proven to have the best performance. Reproduced with permission.^[^ ^71^ ^]^ Copyright 2021, American Chemical Society. d) The multi‐step workflow for the selection of ferromagnetic materials: first, compounds are selected from the C2DB database to construct a training set and test set; next, feature engineering is performed on the datasets, while the GB algorithm is employed to select feature layers and classify materials. Finally, predictions are verified by DFT calculations. Reproduced with permission.^[^ ^81^ ^]^ Copyright 2020, John Wiley and Sons. e) Screening of materials by the GB algorithm. First, 1424 materials with high thermal stabilities are selected from the prediction set; next, ferromagnetic materials are sorted out; finally, the materials are classified as ferromagnetic semiconductors, semimetals, and metals. Reproduced with permission.^[^ ^81^ ^]^ Copyright 2020, John Wiley and Sons.

The XGBoost has achieved a higher level of optimization in engineering practice than GBDT.^[^ ^181^ ^]^ XGBoost supports various types of base classifiers and can automatically learn strategies to handle missing values. Additionally, it incorporates regularization terms when using base classifiers to control model complexity, which helps prevent overfitting and improves the model's generalization ability.^[^ ^2^ ^]^ Indeed, topological insulators (TIs) have been one of the significant topics in quantum materials research.^[^ ^170^ ^]^ Schleder et al. discovered novel 2D topological materials by XGBoost from the databases of C2DB and 2DmatPedia.^[^ ^170^ ^]^ As shown in **Figure** **9**a, they started by gathering the materials, and then used atomic properties as primary features to create an initial dataset. Next, they used SISSO to translate the atomic features into topological classes. Finally, the XGBoost algorithm was used to train a classification model, which was then applied to predict the band topology of 2D materials in more 2D databases. During the training of the RT classifier using the XGBoost algorithm, a penalty is introduced for mispredicted labels train the subsequent tree and correct the errors made by the previous tree. The extreme part of XGBoost has achieved parameter regularization and pruning of the trees, which reduced overfitting and increased the accuracy and scalability of the ML model.

### 4.2 Novel 2D Materials

#### 4.2.1 Catalytic Materials

2D materials, which have a large surface to volume ratio, provide densely distributed sites of surface activities; their excellent mechanical properties can enable durability and thermal conductivity of catalysts, and their feature of electron transfer can directly affect the rate of catalytic reactions.^[^ ^182^ ^]^ As a result, 2D materials have been seeing wider adoption in the development of catalysts. To find efficient 2D water‐splitting photocatalysts, Kumar et al. developed a database of octahedral 2D materials that consists of metals combined with six ligands in an octahedral geometry.^[^ ^65^ ^]^ To increase the speed of the ML model and prevent overfitting, they had to select only the most prominent features, and hence explored the correlations between each feature and each target variable by several ML models (RF, LR, LASSO, recursive feature elimination, XGBoost) to identify the most prominent features. From the aNANt database, they then selected 21 water‐splitting photocatalysts for which the efficiency of HfSe_2_ and ZrSe_2_ reached the theoretical limit. New 2D materials have also been explored in recent years as nitrogen reduction reaction electrocatalysts. Zafari et al. utilized the LGB to predict the adsorption energy of N_2_ and the free energy involved in the intermediate step of the nitrogen reduction reaction.^[^ ^60^ ^]^ By combining DFT and ML, they increased the activity of the catalyst by analyzing the interaction between the active site and the substrate, and found TaB, NbTe_2_, NbB, HfTe_2_, MoB, MnB, HfSe_2_, TaSe_2_, and Nb@SAC to perform the best.

#### 4.2.2 Photoelectric Materials

Exploring new high‐performance 2D photoelectric (2DPE) materials is of great significance in the development of solar cells. Jin et al. devised an efficient method based on ML combined with high‐throughput screening, which employs the latter to discover layered structures from the predicted PE candidates; then, after the removal of the equivalent structures and the validation by DFT calculations, their model identified 26 efficient and accurate 2DPE candidates from the ICSD database, as shown in Figure 9b.^[^ ^71^ ^]^ Moreover, different ML algorithms were employed in the task of prediction, including GB, SVM, RF, Adaboost, stochastic gradient descent classifier, DT, and LR, and their performance was evaluated by four measures—accuracy, recall, precision, and AUC. The result of their study showed that GB outperformed other models (Figure 9c).

#### 4.2.3 Ferromagnetic Materials

There are two main challenges in the development of ferromagnetic 2D materials: small databases and a lack of proper descriptors. To overcome these challenges, Lu et al. constructed a self‐adaptive framework, which generated an iterative feedback loop by combining high‐throughput DFT calculations to enable continuous learning of the ML model; meanwhile, a crystal graph multilayer descriptor was developed based on the crystal graph and elemental properties.^[^ ^81^ ^]^ The multi‐step workflow is shown in Figure 9d. As per the prediction target, they constructed a thermal stability dataset, a magnetic ground state dataset, and a bandgap dataset, and divided the materials in the datasets into three categories: ferromagnetic, antiferromagnetic and non‐magnetic, where 80% of the data was used as a training set, and the remaining 20% as a test set. After identifying the 2D materials data and descriptors, they combined previous research findings and concluded that the GB algorithm exhibited higher performance than other ML algorithms on small‐scale datasets. Therefore, they chose the GB classification algorithm for feature layer selection. By employing this approach, they identified 20 ferromagnetic semiconductors, 21 semimetals, and 51 metals from three databases—C2DB, Materials cloud, and 2D material encyclopedia, with an accuracy rate exceeding 90%, as illustrated in Figure 9e.

In addition, 2D topological magnetic material MnBi_2_Te_4_, characterized by alternating magnetic ordering and a wealth of topological properties, has been extensively investigated for both fundamental and practical purposes. Bhattarai et al. studied magnetic monolayers of the A^i^A^ii^B_4_X_8_ form, building upon MnBi_2_Te_4_, and generated a set of 12360 candidate materials through chemical substitutions at the A, B, and X sites. They subsequently chose an initial subset of 240 structures and determined their formation energy, bandgap, magnetic moment, and magnetic order through DFT calculations. Based on the calculated data, they gained further insight into the microscopic origins of the materials' properties and successfully trained ETR and RFR models to screen 13 promising materials from the candidate materials.^[^ ^85^ ^]^

#### 4.2.4 Other Materials

Tellurene possesses unique properties and advantages over other currently available 2D materials, including a tunable bandgap, high carrier mobilities, and resistance to oxidation.^[^ ^88^ ^]^ Chen et al. designed 385 doped tellurenes by considering 11 types of non‐metal atoms doped on different sites of single‐layer tellurene. By combining first‐principles calculations with an XGBoost model, they investigated the charge transport properties of these materials for potential use in high‐performance electronic and photonic devices, ultimately identifying 23 candidate systems.^[^ ^89^ ^]^ In addition, Zhao et al. employed ML approaches (Pymatgen, FactSage, Aflow and first‐principles calculations) to find 2D TMDs from the Aflow database as Hg^0^ sensing materials to detect and reduce pollutants.^[^ ^161^ ^]^ Fronzi et al. then constructed a dataset of structural properties of 18 million layered vdW structures using the BNN technique based on the 2DMatPedia database, which is designed to facilitate the discovery of novel solid lubricant and super‐lubricant materials.^[^ ^166^ ^]^ There are also publications that probe into the discovery of 2D thermoelectric materials. By comparing different ML models (DT, RF, KNN, MLP and GBDT), Choudhary et al. found that GBDT had the best performance in screening high‐efficiency thermoelectric materials, identifying 128 potential thermoelectric materials from 900 2D materials.^[^ ^169^ ^]^ Kabiraj et al. employed an unsupervised clustering algorithm to find 30 2D materials with potential charge density wave materials from a material database containing >200 “easily‐exfoliable” 2D materials.^[^ ^163^ ^]^

## 5 Preparing 2D Materials

Currently, there is great demand for 2D wafer‐scale films that are compatible with silicon micro‐fabrication techniques in the development of highly integrated devices. By patterning large‐area 2D films into arrays, a series of functional devices can be directly fabricated on a single wafer, ensuring the continuity of highly integrated device structures needed for commercial applications.^[^ ^183^, ^184^ ^]^ Although most 2D layered materials can be obtained by mechanical exfoliation, such method has several limitations, such as low yield, small lateral dimensions of samples, and difficulties in thickness control.^[^ ^185^ ^]^ Other methods, such as liquid phase exfoliation and chemical vapor deposition (CVD), can be applied to the preparation of graphene and some TMDs, but remain challenging to control the layer number, edge shapes, defect densities, and doping densities of the obtained samples.^[^ ^96^ ^]^ Accordingly, ML can be used as an active tool for the preparation of 2D materials, in order to realize the mass production of quality‐controlled 2D devices.

In general, there are two types of preparation methods of 2D materials: one is the bottom‐up method, which is represented by CVD, arc discharge, flash evaporation, exfoliation and deposition/growth, such as molecular‐beam epitaxy and pulsed laser deposition, atomic layer deposition; and the other is the top‐down method, which includes mechanical exfoliation, liquid phase exfoliation and oxidation reduction.^[^ ^2^, ^184^ ^]^ Currently, ML methods for preparing 2D materials are mainly applied in CVD, mechanical exfoliation, and liquid phase exfoliation methods.

### 5.1 Bottom‐Up Preparation

**Figure 10:**
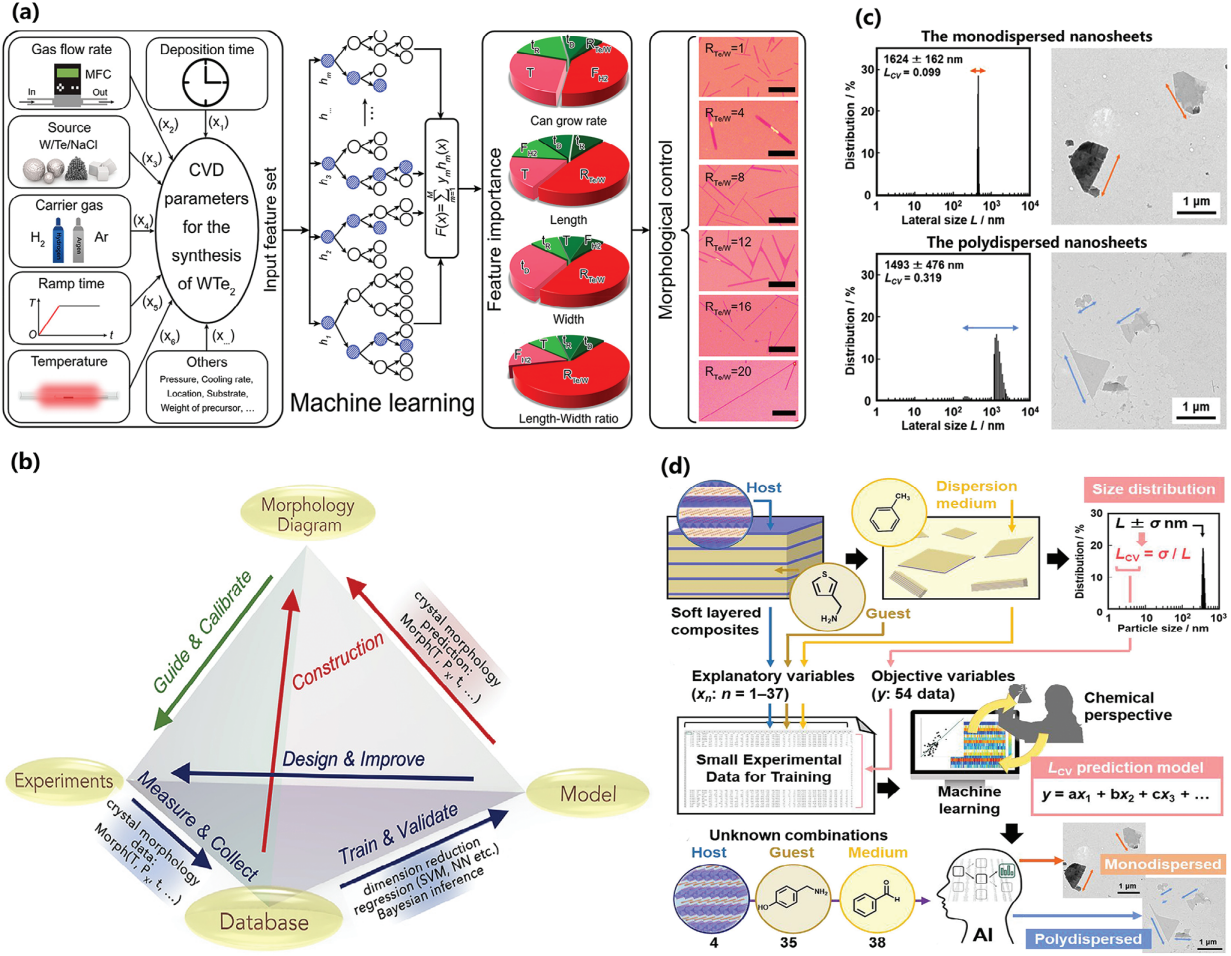
a) Design framework for the realization of geometry‐controlled CVD synthesis of WTe_2_ based on machine learning and experiments. It consists of collecting experimental data, training machine learning models, analyzing experimental parameters, and guiding WTe_2_ synthesis. Reproduced with permission.^[^ ^93^ ^]^ Copyright 2020, Elsevier. b) The relationship between experimentation, modeling, and databases, which is mutually supportive, continuously improves the quality of samples prepared through the CVD method. Reproduced with permission.^[^ ^95^ ^]^ Copyright 2020, John Wiley and Sons. c) DLS charts and TEM images of the monodispersed (DEA)‐Nb_2_O_5_ nanosheets in 2‐propanol and polydispersed (CN‐BA)‐Nb_2_O_5_ nanosheets in water. Reproduced with permission.^[^ ^192^ ^]^ Copyright 2021, American Chemical Society. d) Sparse modeling for size prediction: First, precursor soft layered composites are exfoliated in organic dispersion media; then calculating the size distribution (L_CV_) from the DLS chart and constructing the LCV‐prediction model; finally, AI‐assisted exfoliation experiments. Reproduced with permission.^[^ ^192^ ^]^ Copyright 2021, John Wiley and Sons.

CVD is a process in which the materials in the gas state or the vapor state react on gas‐phase or gas‐solid interfaces to generate solid deposits.^[^ ^183^ ^]^ It performs well in the preparation of 2D thin films, with the size, shape, and thickness all well‐controlled.^[^ ^94^ ^]^ Since the materials that are to be prepared can differ, the experiment conditions for CVD can vary as well, and hence the ML databases in this case are often small databases.^[^ ^93^ ^]^ CVD can be applied to the preparation of high‐quality 2D materials,^[^ ^186^ ^]^ and ML‐enabled CVD preparation of 2D materials includes WTe_2_,^[^ ^93^ ^]^ MoS_2_,^[^ ^94^, ^96^ ^]^ WS_2_^[^ ^97^, ^98^ ^]^ and h‐BN.^[^ ^187^ ^]^ For instance, the trained model proposed by Xu et al. can optimize the CVD synthesis parameters (reaction temperature, rising time, deposition time, airflow rate) to enable controllable growth of multilayer 1D WTe_2_.^[^ ^93^ ^]^ According to ML recommendations, they studied the effect of source ratio (R_Te/W_) on the sample morphology particularly and found the R_Te/W_ dominates the length−width ratio of WTe_2_ NRs in **Figure** **10**a.

Similarly, Xu et al built artificial model functions with three different process windows to mimic actual 2D and 3D thin‐film synthesis and use bayesian optimization to obtain suitable synthetic parameters, such as temperature, oxygen partial pressure, and the sputtering power for thin‐film deposition.^[^ ^188^ ^]^ Li et al. combined self‐organizing maps with means clustering techniques for optical imaging analysis of CVD‐prepared MoS_2,_ which effectively assessed the quality of the CVD‐grown materials.^[^ ^94^ ^]^ Xia et al. developed a DL‐based framework for the analysis of data from kinetic Monte Carlo simulations, and employed KNN, SVM, and RF classifiers to predict the anisotropic growth of WS_2_ monolayers.^[^ ^97^ ^]^

As pointed out in Zhang et al.’s work, the major challenge facing controllable CVD synthesis of 2D materials involves complex correlations between variables in CVD growth, which means that the proper control of these variables is a must to regulate the inherent thermal and dynamic properties of crystal growth.^[^ ^95^ ^]^ To address this problem, they started the Materials Genome Initiative (MGI) to establish morphological diagrams of 2D crystals based on crystal growth experiments, modeling, and databases, as shown in Figure 10b. Meanwhile, to explore the evolution of crystals at different states of growth, they employed ML models (such as SVM, ANN, and decision trees) to unveil the correlations between CVD growth parameters and physical parameters that contribute to crystal growth evolution, thereby predicting crystal growth.

### 5.2 Top‐Down Preparation

Mechanical exfoliation refers to the physical removal of sample layers from layered crystals by applying mechanical forces, such as frictions, to the bulk crystals. This preparation method is both simple and cost efficient, and the obtained samples are often of high crystal quality, with few defects.^[^ ^184^ ^]^ Nonetheless, the size, layers and morphology of the prepared samples are hard to control, and the manual preparation of samples is time‐consuming and inefficient. In this regard, ML‐assisted mechanical exfoliation can achieve automatic identification and classification of exfoliated samples, ultimately leading to large‐scale production.^[^ ^1^ ^]^ Shin et al. designed a fully automated robotic detection system, which combined a graphene trained deep neural network (GT‐DNN) with an optical microscope to classify graphene by size, shape, and thickness.^[^ ^189^ ^]^ Graphene flakes of different layers can be used as target materials of vdW heterostructures, and their findings will also help advance the fabrication of these heterostructures.

In liquid phase exfoliation, graphene is exfoliated from graphite in solvents by using ultrasonic agitation, microwaves, and electrochemical techniques, and the eventual samples are then obtained through centrifugation.^[^ ^190^ ^]^ As with mechanical exfoliation, samples prepared by liquid phase exfoliation are often of high crystal quality and with few defects, but also of a small size and with low yield.^[^ ^184^ ^]^ Moreover, due to the random fragmentation of precursor layered materials during exfoliation, it is difficult to predict and control the lateral size of the exfoliated nanosheets.^[^ ^191^ ^]^ A possible solution to this is sparse ML modeling,^[^ ^191^, ^192^, ^193^ ^]^ which can transform the uncontrolled process of fragmentation into a controlled one.

In sparse modeling, there can be many unknown parameters, but only a few are important and can capture the major features of the regression function, providing an effective solution for modeling high‐dimensional datasets.^[^ ^192^ ^]^ With good explainability, sparse models offer a useful tool for visualizing data, reducing computing overheads and facilitating data storage.^[^ ^193^ ^]^ Haraguchi et al. designed a size‐distribution predictor assisted by sparse modeling (Figure 10d).^[^ ^192^ ^]^ The sparse modeling process they used involved the following steps: first, the precursor layered composites of the host transition‐metal oxides and interlayer organic guests were exfoliated into the surface‐modified nanosheets in organic dispersion media; next, dynamic light scattering (DLS) was used to estimate the lateral size (*L_CV_*) of the transition‐metal oxide nanosheets; finally, potential factors related to *L_CV_* were set as explanatory variables (*x*) and the value of *L_CV_* was set as the objective variable (*y*), which were then input to the ML model to obtain the model parameters. Based on the host‐guest‐medium combinations with single or multiple controlled lateral sizes recommended by the model, they obtained the monodispersed nanosheets by the exfoliation of the layered niobate with intercalation of diethanolamine (DEA) in 2‐propanol and the polydispersed nanosheets by the layered niobate with intercalation of 4‐(aminomethyl)benzonitrile (CN‐BA) in Figure 10c.

## 6 Characterizing 2D Materials

### 6.1 Number of Layers and Thickness

As mentioned above, CVD and mechanical exfoliation are the two currently dominant methods for preparing 2D materials. 2D sheets of different thicknesses that are obtained by these methods often distribute randomly on a substrate, such as SiO_2_/Si or polydimethylsiloxane.^[^ ^109^ ^]^ To study the attributes of different layers of the prepared 2D material, it is crucial to know its exact number of layers. However, the analysis of data obtained by conventional instruments (optical microscopes, Raman microscopes, and atomic force microscopes) relies so heavily on the “intuition” of experienced researchers, making such process both time‐consuming and unreliable. Therefore, several ML models, such as CNN,^[^ ^99^, ^100^, ^101^, ^102^, ^103^, ^104^ ^]^ K‐means clustering (KMC),^[^ ^106^, ^108^ ^]^ SVM,^[^ ^11^, ^110^ ^]^ and RF,^[^ ^111^ ^]^ have been developed to address this issue. Of these models, CNN has unrivalled advantages in terms of image segmentation and object classification, and hence is favored by researchers for identifying the number of layers of atom‐scale sheets on microscopic images.

Trained ML models can extract deep image features, such as optical contrast, RGB, and spectra, to accurately identify the number of layers of 2D sheets. However, challenges still exist in making ML models compatible with microscopic images of different optical settings, while also increasing the model's generalization capacity. Additionally, most databases used for ML model training are small‐scale databases made up of data collected by researchers for specific projects. The following paragraphs discuss the advancements in the ML‐enabled recognition of the number of layers of 2D materials (**Table** **4**).

**Table 4:** Summary of the ML‐assisted characterization of 2D materials. (Full names of the ML algorithms are listed in Appendix).

Research area	2D materials	Preparation method	Source of descriptors	ML algorithms	Assessment mertics	Ref.
Number of layers or thickness	Graphene, h‐BN, TMDs, etc.	Chemical vapor deposition (CVD)	OM images	CNN	Accuracy = 91.0%	[99]
	Graphene	Mechanical exfoliation	OM images	CNN	Accuracy = 99.0% P = 93.5% R = 90.2% F1 score = 0.998	[100]
	MoS_2_	CVD	OM and hyperspectral reflection microscope images	CNN	Accuracy = 97.8%	[101]
	MoS_2_, graphene	Mechanical exfoliation	OM images	CNN	Accuracy = 90.0%	[102]
	MoS_2_	CVD	OM images	CNN	P = 98.6% R = 99.0% F1 score = 0.973	[103]
	Wse_2_, MoS_2_, h‐BN	Mechanical exfoliation	OM images	CNN	Accuracy = 99.9%	[104]
	MoS_2_	CVD	OM images	RCNN	Accuracy >80.0%	[105]
	MoS_2_, graphene	Mechanical exfoliation	OM images	KMC and KNN		[106]
	WS_2,_ MoS_2_, graphene, h‐BN	Mechanical exfoliation	OM images	KMC		[107]
	Graphene	Liquid phase Exfoliation (LPE)	Quantitative polarized optical microscope (qPOM) images	KMC		[108]
	MoS_2_, MoSe_2_	Mechanical exfoliation	OM images	MDC	Accuracy = 95.0%	[109]
	MoS_2_, graphene	Mechanical exfoliation	OM images	SVM		[11]
	Graphene	Mechanical exfoliation	OM images	SVM	P = 98.2% R = 98.8%	[110]
	MoS_2_	CVD	Raman spectroscopy images	RF	P = 99.1% AUC = 0.990	[111]
	Graphene, h‐BN, MoS_2_, WTe_2_	Mechanical exfoliation	OM images	ANN	P≈95.0% R≈97.0%	[112]
	WS_2_, MoS_2_, MoTe_2_, WSe_2_, MoSe_2_, h‐BN, graphene, Bi_2_Sr_2_CaCu_2_O_8_	CVD	OM images	ANN	Accuracy = 96.3%	[113]
Dopants or defects	WSe_2_, MoS_2_, V‐doped WSe_2_, V‐doped MoS_2_	Calculation of QSTEM package simulation	Scanning transmission electron microscopy (STEM) images	CNN	Accuracy≈98.0%	[114]
	2H‐WSe_2−2x_Te_2x_	CVD	STEM images	FCN		[115]
	Mo_1‐x_W_x_Te_2_, WSe_2‐x_Te_x_	CVD	STEM images	CNN		[116]
	Graphene	Calculation of MD simulations	Thermal vibrational morphologies of 2D materials at room temperature	KRR	Accuracy≈95.0%	[117]

**Figure 11:**
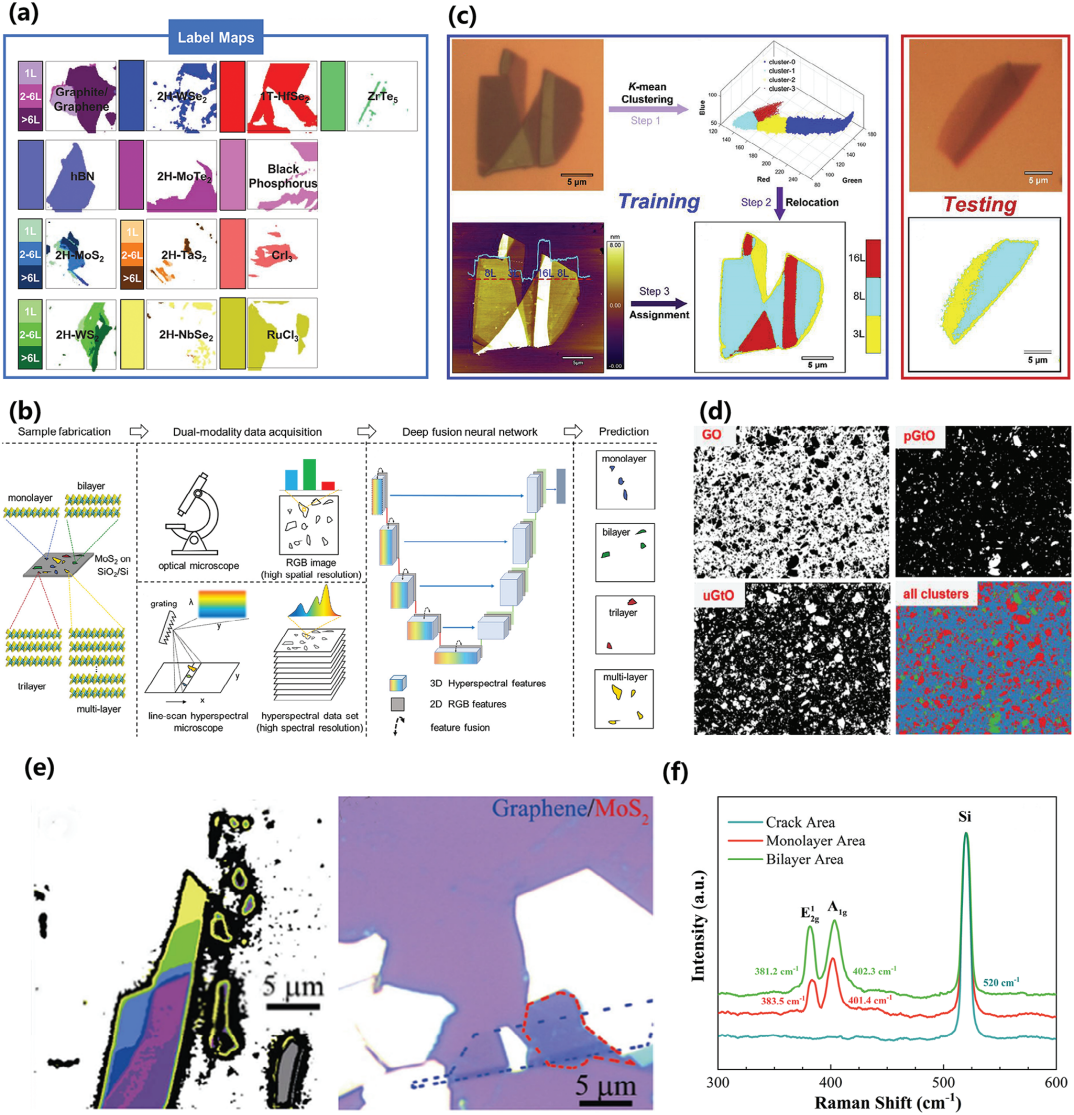
a) The trained CNN model recognized 13 samples and output prediction labels containing the type and thickness of materials. Reproduced with permission.^[^ ^99^ ^]^ Copyright 2020, John Wiley and Sons. b) Atomic layer mapping of 2D materials by 3D‐CNN involves four steps: preparing 2D materials, collecting bimodal data (OM images and hyperspectral images), running DNN training, and predicting layers. Reproduced with permission.^[^ ^101^ ^]^ Copyright 2021, American Chemical Society. c) Diagram of ML‐assisted recognition. In model training, the pixel data, RGB values, and coordinates extracted from the OM image of the 2D material serve as input for the KMC algorithm, enabling the reconstruction of the layer thickness image. In the testing process, the OM images of 2D materials are input to generate the layer thickness detection diagram. Reproduced with permission.^[^ ^106^ ^]^ Copyright 2019, Elsevier. d) Quality detection of LPE‐prepared graphene based on ML, where the uGtO flakes, the pGtO nanoplatelets, and 2D GO sheets represent unexfoliated, partially exfoliated, and well‐exfoliated species, respectively. Reproduced with permission.^[^ ^108^ ^]^ Copyright 2020, John Wiley and Sons e) SVM‐enabled detection of layers of MoS_2_ samples (different colors indicate different layers), as well as graphene and MoS_2_ vertical heterostructures (graphene and MoS_2_ are indicated by blue and red dashed lines, respectively). Reproduced with permission.^[^ ^11^ ^]^ Copyright 2019, American Chemical Society. f) Raman spectra of monolayer, cracks, and bilayer areas of MoS_2_ samples, where the spectral information is used as input features to distinguish different areas. Reproduced with permission.^[^ ^111^ ^]^ Copyright 2020, Multidisciplinary Digital Publishing Institute.

In Han et al.’s work, a CNN was utilized to study the optical microscopic (OM) images of 13 materials, which extracted features, such as RGB, edge, shape, and size to identify the types and thicknesses of the 2D materials.^[^ ^99^ ^]^ In addition, six types of OM images were collected to increase the CNN model's generalization capacity. They used the random‐rotation augmentation method to augment the data, which produced random positioning and orientation of images. The trained CNN model could identify individual flakes and distinguish both the material identities and thicknesses of the 13 2D materials with a high success rate (**Figure** **11**a). It was also found to be robust against variations in features, such as brightness, contrast, white balance, and non‐uniformity of the light field. However, identifying the number of atomic layers remains challenging, since the RGB images of atomic‐layer flakes are often similar in appearance. In response, Dong et al. merged hyperspectral reflection images and RGB images to identify and split MoS_2_ flakes prepared by CVD, and augmented the dataset by random rotation and flip.^[^ ^101^ ^]^ They employed a 3D convolutional neural network (3D‐CNN) to segment and recognize mono‐, bi‐, tri‐, and multilayered MoS_2_ flakes. Figure 11b shows how the atomic‐layer mapping of 2D materials is enabled by the ML model. Their approach substantially reduced the time needed for data collection and pretreatment, and predicted layer distributions and segments individual layers with a significantly higher level of accuracy (>80%) than conventional RGB methods (∼60%). Moreover, their method was experimentally proven to have a strong robustness against illuminating and contrasting variations.

KMC has also been popular in imaging and spectral analysis. Li et al. combined Fresnel law and ML for OM imaging analysis, using optical contrast, total color difference, and RGB to find the optimal substrate and recognize the layers of 2D materials.^[^ ^106^ ^]^ In their work, the KMC algorithm was employed to study subtle color differences between images of different layers, and thereby to construct a database of 2D materials layers (Figure 11c); the KNN algorithm was then used for testing to achieve automatic recognition of the layers of samples. They found that the optimum Si/SiO_2_ substrate thicknesses for mechanically exfoliated graphene and MoS_2_ were 90, 100, 270, and 300 nm for the oxide layer, and their model could reach a certain accuracy in detecting these four layers. On the contrary, conventional OM has difficulty in identifying the size and thickness of few‐layer graphene, due to its low photon absorption capacity. To address this challenge, Abedin et al. put forth a quantitative polarized optical microscope for capturing birefringence images of graphene dispersions.^[^ ^108^ ^]^ By using contrasts in the bright‐field and cross‐polarized optical features, they applied the KMC algorithm to study the thickness of the graphene prepared by LPE. This technique was best suited for samples containing nanoplatelets and flakes with a total concentration between ≈0.02 and 2 wt.% solids. This method identified effectively three different data clusters representing flakes (unexfoliated), nanoplatelets (partially exfoliated), and 2D sheets (well‐exfoliated) species in various dispersions of graphene and graphene oxide (Figure 11d).

SVM is particularly suitable for analyzing small samples. Lin et al. extracted RGB information in OM images of exfoliated graphene and MoS_2_ to characterize the thicknesses and stacking orders of their 2D heterostructures (Figure 11e).^[^ ^11^ ^]^ In their work, the images were pretreated to improve the model's generalization capacity, and the treatment included de‐noising and color calibration. In addition, the RF algorithm is capable of analyzing multiple Raman features to identify samples, addressing the limitation of identifying samples using only a single variable. Mao et al. utilized the RF algorithm to extract two features, peak intensity and frequency information, from spatial mapping of Raman spectra.^[^ ^111^ ^]^ These two features were used as inputs, and the sample thickness and type as outputs to generate decision trees for the classification of monolayer MoS_2_ continuous film, random cracks, and bilayer areas (Figure 11f).

### 6.2 Defects

Structural defects and foreign atoms can substantially impact the performance of 2D materials. However, accurately determining the distribution and local concentration of these defects and atoms with picometer‐level precision remains a challenge. While scanning transmission electron microscopy (STEM) provides an imaging solution for single atoms, its accuracy is limited to the signal‐to‐noise ratio. High‐dose radiation enables the precise measurement of individual atom positions, but this will incur changes in defective 2D structures because of ionization effect. Low‐dose irradiation, however, obtains images with high noise levels, thus failing to enable the quantitative evaluation of atomic defects.^[^ ^115^ ^]^ Here, an ML‐enabled solution is to rapidly collect a series of images from the same area under low‐dose irradiation and employ the CNN algorithm to augment and de‐noise the images in order to reach a higher signal‐to‐noise ratio.^[^ ^114^, ^115^, ^116^ ^]^ Table 4 highlights relevant publications in this regard.

**Figure 12:**
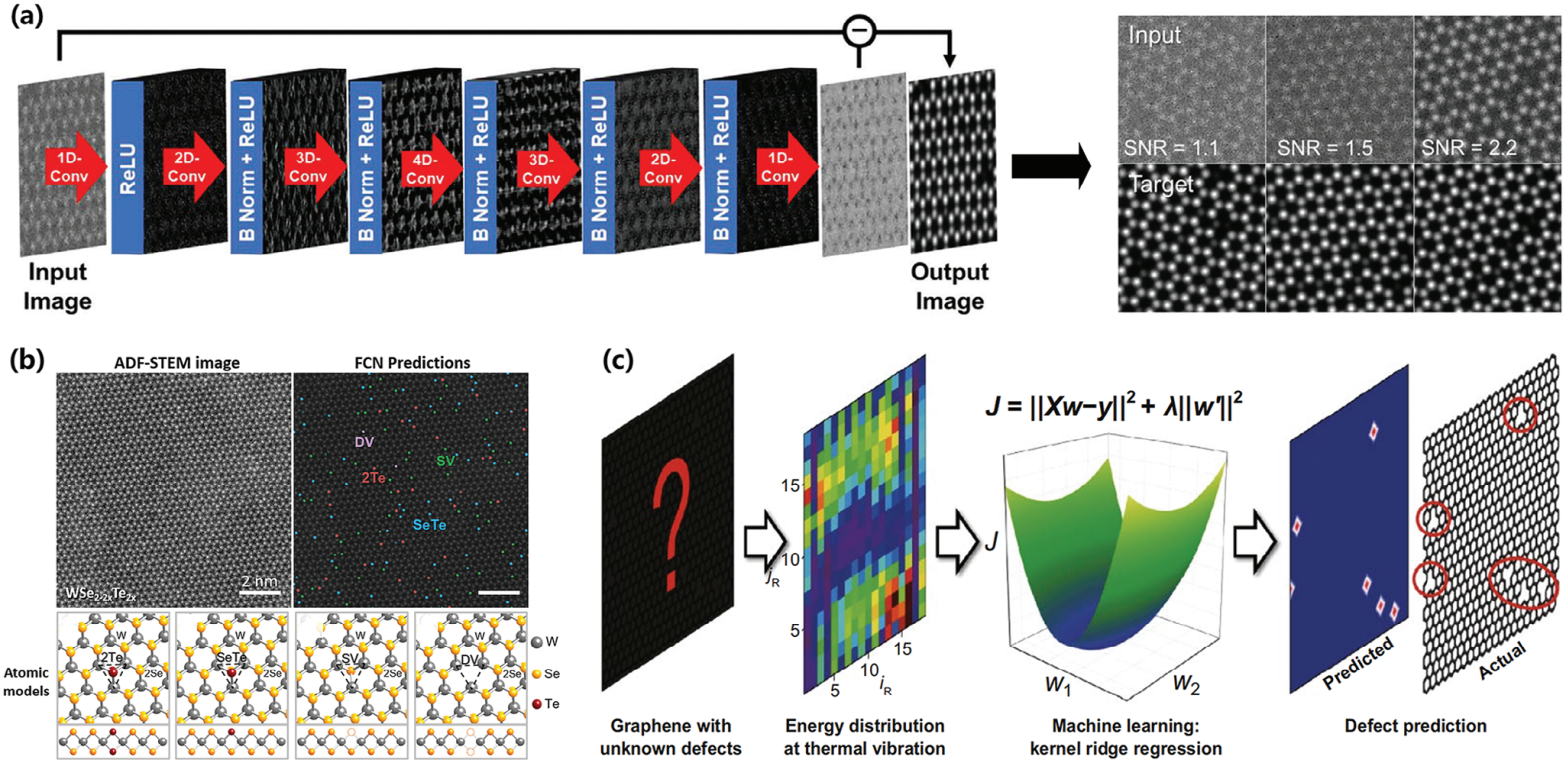
a) A DNN model built to de‐noise ADF STEM images of V‐WSe_2_: statistical noise backgrounds are extracted from ADF images as independent signal features, which are then removed from the input images to restore the actual atomic contrast of images. Reproduced with permission.^[^ ^114^ ^]^ Copyright 2021, John Wiley and Sons. b) DL‐enabled recognition and classification of defects in ADF‐STEM images, including ADF‐STEM images of WSe_2−2x_Te_2x_ and maps of defects of FCN‐detected chalcogen‐site defects. Reproduced with permission.^[^ ^115^ ^]^ Copyright 2020, American Chemical Society. c) ML‐enabled prediction of graphene defect positions. The KRR model extracts energy distribution features of graphene under thermal vibrations to predict the sites of defects. Reproduced with permission.^[^ ^117^ ^]^ Copyright 2020, Springer Nature.

Yang et al. have put forth a DL‐assisted model for classifying and locating atomic dopants and defects in 2D TMDs, such as WSe_2_, MoS_2,_ V‐doped WSe_2_ and V‐doped MoS_2_.^[^ ^114^ ^]^ The annular dark‐field (ADF) STEM images in their study were generated through multi‐layer computational simulations by using the QSTEM software package. CNN‐based image restoration techniques were employed to reduce noises and enhance the contrasts of the STEM images (**Figure** **12**a). Also, a fully convolutional network (FCN), which shows an excellent performance in the segmentation of image features, was designed to achieve reliable quantification of dopants and defects in TMDs with single‐atom precision. Based on experimental observations, they classified the possible atomic sites into five different types: W, V substituting for W, Se with no vacancy, mono‐vacancy of Se, and di‐vacancy of Se. Moreover, their ML approach demonstrated that atomic dopants and defects were precisely mapped with a detection limit of ≈1 × 10^12^ cm^−2^, and with a measurement accuracy of ≈98% for most atomic sites. Lee et al. synthesized 2H‐WSe_2‐2x_Te_2x_ samples via cooling‐mediated CVD on SiO_2_/Si substrates, which suffered from defects, including Te substitutions and Se vacancies.^[^ ^115^ ^]^ In their work, a dataset of aberration‐corrected ADF‐STEM images was created, where STEM images were acquired as ten sequential frames with short dwell times (2 µs pixel^−1^) in the same region and then frame‐averaged to minimize image distortions from sample drift and reach an accuracy of 0.2 pm in measuring 2D interatomic distances. They used a FCN‐based DL model to locate and classify the point defects and generate a 2D map for defects, including the four primary types of chalcogen‐site defects, as shown in Figure 12b.

Other approaches have also been developed to characterize point defects of 2D materials other than from STEM. Zheng et al. employed the kernel ridge regression (KRR) algorithm to explore the hidden correlations between the defect points and thermal vibration.^[^ ^117^ ^]^ The KRR model, which was trained by thousands of thermal vibration morphologies computed by MD simulations, could accurately detect uniformly dispersed defects of graphene (Figure 12c). They proposed two prediction strategies: one based on atomistic approach that constructs data by atom indexing, and the other based on domain approach that constructs data by domain discretization. The results indicate that the atomistic approach can predict single atomic vacancies, while the domain approach can accurately predict multiple vacancies of unknown quantity. Both methods can achieve a prediction accuracy of about 90% on the retained test data.

## 7 Guiding Fundamental Research on 2D Materials

In addition to the advances mentioned above, ML has also contributed greatly to fundamental research on 2D materials, as summarized in **Table** **5**.

**Table 5:** Summary of ML‐guided fundamental research on 2D materials. (Full names of the ML algorithms are listed in Appendix).

Research area	Materials	ML algorithms	Assessment mertics	Ref.
Uncovering the effect of atomic‐scale defects on the properties of 2D materials	TMDs, h‐BN	DNN	MSE = 0.010 MAE = 0.060 R^2^ = 0.980	[194]
Exploring the role of point defect distribution in inducing phase transitions in monolayer 2D materials	MoS_2_	GA		[195]
Searching for the best defect configuration with the lowest energy	MoS_2_	RL		[196]
Investigating the impact of the distribution of holes on the thermal conductivity of graphene	Graphene	CNN	RMSE = 1.090 R^2^ = 0.970	[197]
Investigating the electronic properties of 2D coordination polymer (CPs) adsorption on germanium and silica	2D CPs	ANN		[198]
Unfolding the structure‐property relationships of Li_2_S anchoring on 2D materials	2D A_x_B_y_ (B in the VIA/VIIA group)	GBR	RMSE = 0.935 R^2^ = 0.998	[199]
Discovering and designing novel 2D horizontal interfaces	Blue phosphorene	GNN	MAE = 1.010	[200]
Finding the optimal design of the Kirigami structure	MoS_2_	RL		[201]
	Graphene	CNN	RMSE = 0.053 R^2^ = 0.920	[202]
Identifying the twist angle of bilayer graphene using Raman spectroscopy	Twisted bilayer graphene (tBLG)	KRR, RFR and MLP	MLP MAE≈0.270 RMSE≈0.690 R^2^≈0.980	[203]
	tBLG	RF	Accuracy≈99.0%	[204]
Revealing the correlation between the formation and exfoliation energies of the MAB phase and structure	2D transition‐metal borides (MBenes)	SVM, DNN, and RFR	RFR MAE = 0.040 R^2^ = 1.000	[13]
Predicting low‐temperature exciton valley polarization	WSe_2_	RF		[205]
Detecting the nanopores in 2D materials by TEM images	1T‐CrTe_2_	DL		[206]
	MoS_2_, WS_2_	GB	Accuracy = 96.0%	[207]
Employing automated scanning probe microscopy driven by hypothesis learning to investigate bias‐induced switching in ferroelectric materials	Ferroelectric materials	BO		[208]
Predicting thermally and mechanically induced ripples	Graphene, h‐BN	GAP	RMSE = 0.003	[209]
Exploring the electronic properties of arbitrary layered materials	MoS_2_, graphene	NN	R^2^ = 0.630	[210]
Exploring the optimal thickness of 2D materials as diffusion barriers for copper	Graphene, h‐BN	CNN	MSE = 0.008 MAE = 0.070 RMSE = 0.090 R^2^ = 0.999	[211]
Finding descriptors affecting frictional properties of 2D materials	Graphene, TMDs	TL		[212]
Optimizing Process Conditions for Top‐Gate Field Effect Transistor (FET)	MoS_2_	EL and RFR		[14]
Exploring some information about FET devices through the LF noise spectrum	MoS_2_	CNN	Accuracy = 95.5% F1 score = 0.930 AUC = 0.830	[213]
Finding the Optimal Conductive Sensing Materials for Manufacturing Pressure Sensors	Hybridization of 2D Ti_3_C_2_T_x_ MXenes with 1D nitrogen‐doped graphene nanoribbon	ANN	Accuracy = 97.2%	[214]
Investigating the variation of the two‐phase characteristic in nanoribbons of different widths	Graphene	MLIPs	RMSE = 0.004	[215]
Exploring parameters affecting graphene mechanical properties	Graphene	ML		[216]
Unraveling the correlation between Raman and photoluminescence	MoS_2_	DenseNet, XGBoost, and SVM		[217]
Unveiling the dependence of laser power and temperature on Raman shifts of 2D TMDs	MoS_2_	LR, DTR, and RFR	MSE = 1.97 × 10^−9^ RMSE = 4.44 × 10^−5^ R^2^ = 0.990	[218]
Addressing the challenge of incomplete annotation for instance segmentation in the identification of 2D quantum materials	2D materials	DL		[219]
Detecting the existence of crystal line defects in samples by analyzing raw 2D coherent diffraction data.	FCC materials	CNN	Accuracy = 95.4%	[220]
Exploring the configurational space of amorphous graphene	Amorphous graphene	GAP		[221]
Exploring the best structure of hydrogenated graphene	Hydrogenated graphene	XGBoost	RMSE = 0.033 R^2^ = 0.980	[222]
Assessing the feasibility of exfoliating any 3D compound into 2D layers	2D materials	RF	P = 88.0% R = 98.0% F1 score = 0.930	[15]
Investigating the flexoelectric energy conversion in bilayer vdW structures	2D vdW bilayers	MLIPs		[223]
Simulating Raman spectra of titanium carbide MXenes using ML algorithms	Titanium carbide MXenes	ML		[224]
Investigating the ferroic phase transformation in monolayer GeSe nanoribbons	GeSe	MLIPs		[225]
Detecting the strain in TMDs by Raman Spectroscopy	WS_2_	KMC		[226]
Predicting the interlayer sliding energy barrier of MoS_2_ layers	MoS_2_	LR	MSE = 0.002 R^2^≈1.000	[227]
Capturing 2D vdW magnets with with a high likelihood of experimental realization from materials science literature	2D materials	ANN		[16]
Reconstructing the exit wave of 2D materials in high‐resolution transmission electron microscopy	2D materials	CNN	RMSE = 0.006	[228]
Generating extreme quantum scattering in graphene	Graphene	ANN		[229]

**Figure 13:**
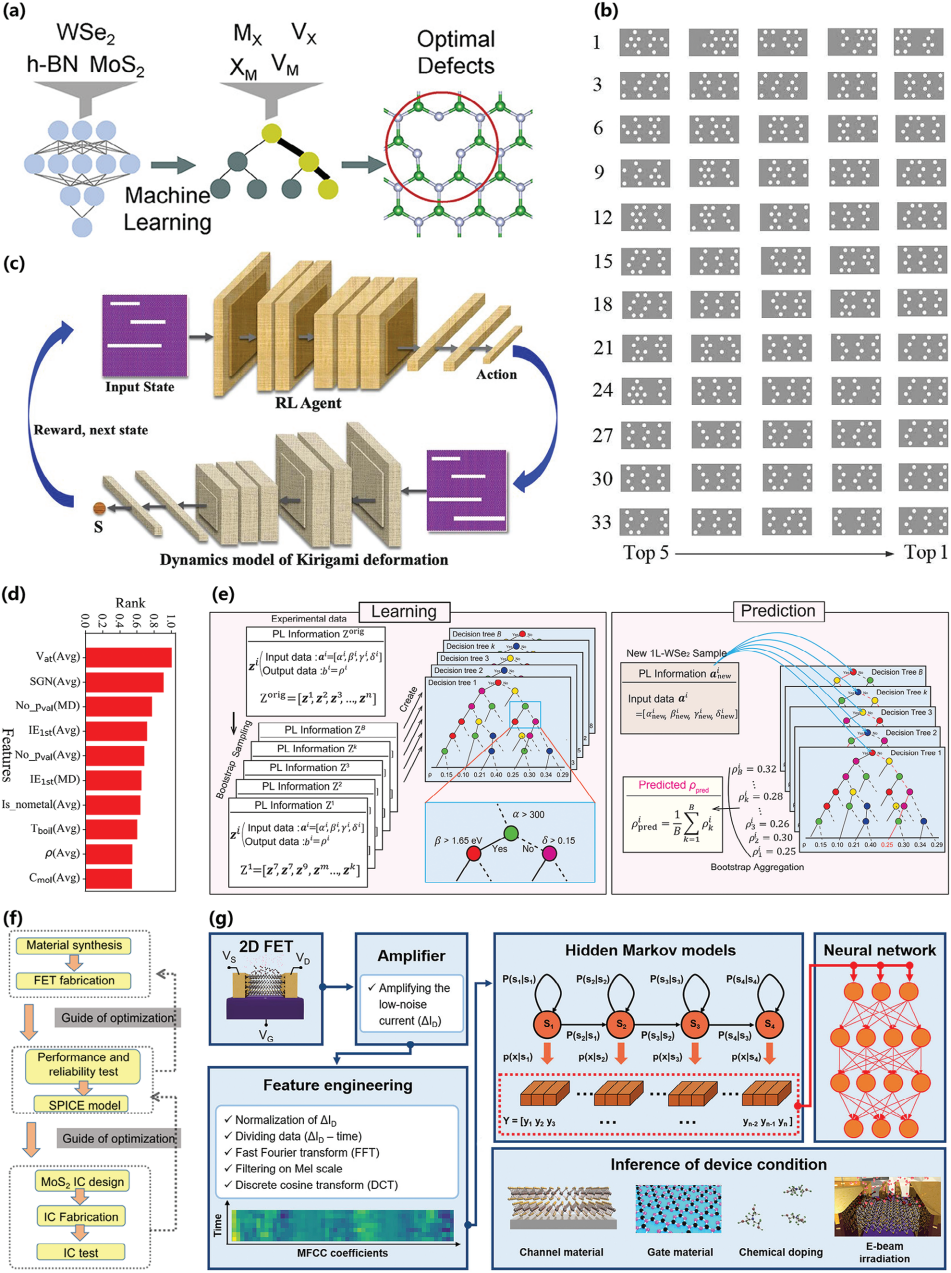
a) Workflow of ML‐based prediction of ideal candidate defects. Deep transfer learning detects deep‐center defects of 2D materials, while the RF model predicts structural characteristics of the defects in order to obtain the ideal type of candidate defects. Reproduced with permission.^[^ ^194^ ^]^ Copyright 2020, American Chemical Society. b) Five porous graphene structures with the lowest thermal conductivity predicted by CNN. Reproduced with permission.^[^ ^197^ ^]^ Copyright 2020, Elsevier. c) The ML model that predicts MoS_2_ kirigami structures with high tensibility: the model predicts tensibility based on the agent and provides rewards, with the agent using these rewards to update its strategy to maximize the total reward and thereby construct structures of higher tensibility. Reproduced with permission.^[^ ^201^ ^]^ Copyright 2021, Springer Nature. d) The ten most important features affecting MAB‐phase exfoliation. Reproduced with permission.^[^ ^13^ ^]^ Copyright 2020, American Chemical Society. e) RF module: each decision tree has several nodes, and the threshold values of variables at each node are computationally determined to maximize the amount of information gained. This approach is designed to improve the accuracy of replicating the low‐temperature exciton valley polarization landscape of monolayer WSe_2_. Reproduced with permission.^[^ ^205^ ^]^ Copyright 2019, American Chemical Society. f) Application scene of ML‐assisted fabrication of 2D FETs, from material synthesis to industrial circuit design, fabrication, testing, feedback, and optimization. Reproduced with permission.^[^ ^14^ ^]^ Copyright 2021, Springer Nature. g) Feature learning and classification of 2D FETs: the ML model extracts features of FET current signals measured by a low‐noise current amplifier; these features are then engineered into digital features recognizable by the hidden Markov model‐based neural network to infer the conditions of devices. Reproduced with permission.^[^ ^213^ ^]^ Copyright 2021, Springer Nature.

In recent years, there have been several ML‐based studies exploring the impact of atom‐scale defects,^[^ ^194^, ^195^, ^196^ ^]^ doping,^[^ ^197^ ^]^ and adsorption^[^ ^198^ ^]^ on the performance of 2D materials. Based on C2DB database and DFT calculations, Frey et al. utilized the ML technique to identify the top 100 deep center defects suitable for quantum emission and the ten best defects for nonvolatile resistive switching in atomically thin memristor devices.^[^ ^194^ ^]^ Their ML approach consists of two models: a DL classifier that predicts center defects, and a RFR model that predicts energy differences between the defective structure and the pristine host structure (**Figure** **13**a). The ML model uses easily accessible descriptors and dispenses with electron structure calculations to code local relaxation and capture electronegativity. Wan et al. employed a CNN model to unveil the impact of hole distribution on the thermal conductivity of monolayer graphene.^[^ ^197^ ^]^ Through the MD simulations, they generated 10^3^ different structures to identify the most effective distribution of holes for reducing the thermal conductivity of porous graphene (Figure 13b). The most effective pore distribution for achieving the lowest thermal conductivity in porous graphene, as revealed in their work, involves transverse pores exhibiting periodicity along the direction of heat flow. This spatial distribution led to the localization of phonon modes, resulting in a decrease in thermal conductivity. Based on DFT calculations and big data mining, Shayeganfar et al. combined the neural network and maximum likelihood analysis to explore the electrical properties of 1D and 2D coordination polymers adsorbed on graphene and SiO_2_.^[^ ^198^ ^]^ Their work found that states mixing and small charge transfer between the graphene and adsorbate, provoked a slight bandgap opening by breaking the local symmetry of graphene band states, which led to the changes in their structural and electronic properties. Their discovery opened up a new category of materials with unique electronic surface states and provided a foundation for developing various inorganic heterostructures.

The tensile properties of 2D materials^[^ ^201^, ^202^ ^]^ can be regulated by using cutting or inserting techniques like the kirigami craft. Rajak et al. generated a diverse set of MoS_2_ kirigami structures with high stretchability by using a RL model (Figure 13c), where the stretchability is defined as the maximum strain the material can withstand without failure.^[^ ^201^ ^]^ By training the RL model on data from MD simulations based on three variables—the total number of cuts, the cut locations and lengths—they showed that a system consisting with up to 6 cuts could achieve a maximum stretchability exceeding 45%. Additionally, vibrational properties of twisted bilayer graphene are characterized by complex features that arise from its intricate energy landscape in low‐symmetry configurations. By using dimensionality reduction and a decision‐tree based regression, and with the computational Raman spectra of numerous twisted bilayer graphene structures as input features, Sheremetyeva et al. established the relationship between the twist angle of a graphene bilayer structure and the Raman intensities, with the intensity profile near the calculated G‐band identified as the most significant feature.^[^ ^203^ ^]^ MAB phases have been applied for the exfoliation of 2D transition metal borides (Mbenes), which possess great potential in the development of advanced nanodevices. Using three ML models—RFR, DNN, and SVM—Siriwardane et al. explored the relationship between the structural factors, exfoliation energy and formation energy of MAB phases with hexagonal and orthorhombic crystal symmetries.^[^ ^13^ ^]^ They utilized the pymatgen python module to obtain the formation energies of 7000 materials from the Materials Project database, and screened the ten most important features (Figure 13d) to train and test the ML models. They found that the formation energy of MAB phases could be turned by adjusting the A element, with a higher atomic number of A corresponding to less stability in the MAB phase and easier exfoliation of 2D MBenes. Recent attention has been drawn to the potential applications of TMDs in future optoelectronics, but predicting the low‐temperature heterogeneity of exciton valley polarization solely from room temperature measurements is challenging. Tanaka et al. used a RF model (Figure 13e) to extract information from the room‐temperature photoluminescence spectra of monolayer WSe_2_ to successfully predict the low‐temperature exciton valley polarization landscape.^[^ ^205^ ^]^ They found that variables related to the exciton intensity and carrier density were the key factors that determined the local exciton valley polarization.

While ML has facilitated the transition of 2D materials research from theoretical exploration to practical application, challenges remain in achieving high‐quality growth and circuit‐level integration. To overcome these challenges, Chen et al. utilized ML models to analyze the experimental data of wafer‐scale fabrication of 2D MoS_2_ top‐gated field‐effect transistors (FETs) and assess the crucial process parameters that affect the electrical properties, and therefore optimize the fabrication technique and improve the electrical performance of FETs.^[^ ^14^ ^]^ In their work, the ML models they used included a decision tree‐based ensemble learning model and a RFR model; the analyzed data was correlated to the device performance, including current on‐off ratio, threshold voltage, carrier mobility, and sub‐threshold swing. After optimizing wafer‐scale material and device‐fabrication processes, they moved on to device characterization, SPICE modeling, and circuit design. To accomplish this, they used industry‐standard design flows to create wafer‐scale test FET arrays and a 4‐bit full adder (Figure 13f). Their findings revealed the huge potential that ML has in assisting and optimizing the fabrication of electronic materials beyond silicon. Low‐frequency (LF) *1/f* noise spectroscopy is a nondestructive defect diagnosis tool which identifies dominant scattering origins that are caused by phonon vibration, the Schottky barrier inhomogeneity, interlayer resistance, imperfect crystallinity, and traps inside the materials and dielectrics. Lee et al. developed an efficient and accurate method for characterizing and classifying layered 2D FETs by combining LF noise spectroscopy with a neural network based on hidden Markov models.^[^ ^214^ ^]^ Their ML model could classify important information of devices based on >100 LF noise data sets under 32 conditions, such as gate dielectrics, type of channel materials, and contact metals (Figure 13g).

## 8 Conclusion and Prospects

As this review shows, ML has significant potential in accelerating the development of 2D materials. Traditional techniques, such as DFT calculations and classical MD simulations alone, cannot handle massive amounts of data. They also require high levels of performance in hardware. In contrast, ML not only reduces these computing overheads, but also surpasses traditional calculation methods in accuracy. ML models trained by databases consisting of DFT calculation results and experiment statistics have been widely used in research on 2D materials: characterizing the layers and defects of materials, identifying preparation conditions, predicting properties, and developing new 2D materials, while much research primarily focus on these four areas. ML has also played an exceptional role in the fundamental research on 2D materials, such as the correlation between thermal conductivity and hole distribution of monolayer graphene,^[^ ^197^ ^]^ optimal design of kirigami structures,^[^ ^201^, ^202^ ^]^ and analysis of technical parameters that affect electrical properties of top‐gated 2D MoS_2_‐based FETs.^[^ ^211^ ^]^ Some relevant publications are listed in Table 5. In summary, this review discusses the latest progress of applying ML in 2D materials, and summarizes the commonly used algorithms, descriptors, and workflows of ML in exploring different research scenarios of 2D materials. The cross‐combination of ML and 2D materials has not only presented new challenges but also brought new opportunities.

For ML‐enabled prediction of properties of 2D materials and development of new 2D materials, using an extensive array of chemical, structural and other initial features is necessary for training a ML model. However, if these initial features are not preprocessed with existing physical or chemical knowledge, or one relies solely on ML algorithms to handle the intricate relationships among them, reduced computational efficiency and minor prediction inaccuracy of the model may occur.^[^ ^80^, ^124^ ^]^ Descriptors refactoring provide a solution to this problem, these initial features can be restructured into new features, based on specific correlation functions or new theoretical constraints, which not only expand the number of samples, but also improve the ML model's fitting capacity to include complex relations between features. This method of creating descriptors based on combinations of features has played a crucial role in predicting the properties of 2D materials, developing new 2D materials, and guiding fundamental research on 2D materials.^[^ ^33^, ^170^, ^210^ ^]^ Theoretical explanations of the correlations between different descriptors and target property are also necessary to give researchers a more in‐depth, theory‐based understanding of the target properties and improve the ML model's prediction efficiency and accuracy. In addition, interpretable ML models rooted in physical or chemical knowledge can instill reliability in model predictions and unveil unexpected correlations that may lead to scientific insights into physical chemistry.^[^ ^230^ ^]^ MLIPs algorithm involve the creation of interatomic potentials through ML model training.^[^ ^125^ ^]^ The crystal diffusion variational autoencoder method enables direct learning of material properties from the atomic connections within the crystal.^[^ ^30^ ^]^ Furthermore, the application of SHAP analysis to the RF model helps us understand how physical descriptors contribute to the model's predictive capacity for the magnetic ordering of 2D materials.^[^ ^137^ ^]^

Although there are some established open‐source databases of 2D materials, such as C2DB, 2DMatPedia, and Materials Cloud, these databases are still far from sufficient for ML‐enabled interdisciplinary research on 2D materials. As shown in the tables associated with Sections 4 and 5, many publications on 2D materials still rely on small‐scale databases created specifically by researchers through DFT calculations and MD simulations, the findings from which are limited. Furthermore, When the dataset is insufficient in size, it can give rise to overfitting or underfitting in ML models. Overfitting occurs when a ML model overly fits the training data, meaning it learns the noise and specific examples within the training data, resulting in strong performance on the training data but poor generalization and application performance. Conversely, underfitting occurs when a ML model fails to capture the genuine patterns and relationships within the data, leading to subpar performance on both the training and test data. However, 2D materials and their associated properties developed based on ML can be verified by DFT calculations, experiments and multiple ML models.^[^ ^91^ ^]^ Once verified, these new data can be updated or added to existing open‐source databases, which can then be reapplied to train ML models and accelerate advances in research on 2D materials. For instance, Thygesen et al. utilized two different methods (eigenvalues and wave functions) to generate features of individual electronic states, which were then employed to train the ML model for predicting the G_0_W_0_ corrections to the PBE band structures of ≈700 2D semiconductors from the C2DB. These band structures have been published on the C2DB web page.^[^ ^127^ ^]^ For the time being, databases of image processing‐based layers and defect characterization data exist independently of one another, which makes it hard to obtain enough data for ML model training. In this framework, the FAIR principles (Findability, Accessibility, Interoperability, and Reuse) are becoming a recognized standard for open‐access data availability and reuse, with univocal metadata definitions currently under definition among the different scientific communities.^[^ ^231^, ^232^ ^]^ In the future, efforts should be made to build more complete image databases, and database users should be encouraged to upload their experimental images to achieve massive amounts of data collection. Furthermore, while ML has shown promise in accelerating 2D materials research, it is important to note that models are usually trained using single algorithms. Since first‐principles calculations are unable to handle massive amounts of data, it becomes challenging to measure the deviation of the ML predictions from the actual values. To address this challenge, it is essential to train the model using two or more different algorithms, yielding predictions separately. By comparing these predictions, the best algorithm for model training can be identified.

In addition, the family of 2D materials has expanded tremendously over the last decade, collectively encompassing a huge portfolio of properties ranging from semiconducting and insulating to magnetic, and superconducting. Emerging members include layered oxides with atomic layers connected by oxygen bridges, weak covalent bonds, or intercalating elements,^[^ ^233^, ^234^ ^]^ in turn endowing them a high degree of chemical tunability via functionalization,^[^ ^235^ ^]^ alloying,^[^ ^236^ ^]^ doping,^[^ ^237^ ^]^ intercalation of ions and molecules,^[^ ^238^ ^]^ etc.; the possibilities for property‐oriented materials design are thus immense. Nevertheless, the most enduring impact of 2D materials in terms of innovative devices and architectures is expected to come from their heterostructures.^[^ ^239^ ^]^ In this case, electrons in atomically thin 2D layers are exposed to layer‐to‐layer coupling, allowing for the interaction and coupling of different properties in the individual layers in ways that are otherwise impossible in other systems.^[^ ^240^, ^241^, ^242^ ^]^ The coupled properties not only are highly exotic by themselves but also can be modulated by various means, such as the combination of constituent materials, stacking sequence^[^ ^243^, ^244^, ^245^ ^]^ and relative crystallographic alignment.^[^ ^246^ ^]^ Moreover, a plethora of extended opportunities has been revealed in vdW heterostructures that are created by incorporating molecular species, such as molecule‐intercalated 2D layered crystals, organic‐2D interfaces, as well as mixed 2D/3D heterostructures.^[^ ^247^, ^248^, ^249^, ^250^, ^251^, ^252^ ^]^ These are just non‐exhaustive examples for capturing the great potential of 2D materials for realizing materials by design. The 2D Crystal Consortium^[^ ^253^ ^]^ and Brookhaven National Laboratory's Quantum Material Press (QPress)^[^ ^254^ ^]^ are collective platforms committed to the design and preparation of 2D materials and layered heterostructures.

However, with vast material choices and enormous parameter space at our disposal, this also implies a must to collect and sift through an immense amount of complex data to identify meaningful information. In tandem with this challenge, ML enables efficient and accurate algorithm‐based approaches for accelerated discovery and intelligent design of 2D materials and heterostructures. Autonomous experimentation is another ML‐enabled aspect, which is essential in both reducing the number of experiments to a more tractable scale and accelerating the process of making decisions to match the rate of incoming data. Within the scope of 2D materials, most attempts toward this end are only semi‐autonomous, i.e., mainly limited to characterization tasks, such as identifying layer number and atomic defects (see Sections 4 and 5), in which decision‐making still relies heavily on human intervention. However, true autonomy should become reachable when a full algorithm‐driven cycle is developed for the processing‐structure‐properties‐performance relationships. In particular, we expect this to create a long‐lasting impact on 2D materials research that employs large‐scale facilities, notable examples of which include synchrotron radiation light sources^[^ ^255^, ^256^, ^257^, ^258^ ^]^ and large‐scale material fabrication and analysis platforms, such as Le TUBE‐Daum at the Jean Lamour Institute in France,^[^ ^259^ ^]^ and Nano‐X in China.^[^ ^260^ ^]^ These strategically built facilities have integrated capabilities for material growth, characterization, and post‐growth processing. Multiple experimental stations are installed as an all‐in‐one system and connected through ultrahigh vacuum pipelines to achieve comprehensive studies of materials in a contamination‐free environment. To this end, NANO‐X has, for instance, planned 500 meters of pipelines and ≈100 stations, of which 44 are already in place. While these facilities have been deployed to great effect for low‐dimensional materials, it remains a daunting task to map out the right conditions at each station for the best possible data output, which could vary between users and even take months to master. In aggregate, these issues are expected to motivate the establishment of an automated control framework for large‐scale facilities, that will integrate ML algorithms at multiple scales to perform not only equipment control but more ambitious tasks, such as scientifically relevant modeling, interpretation, and uncertainty quantification of multiple streams of incoming data.

The takeaway message this review aims to deliver – ML is incurring revolutionary shifts in 2D materials research. Moreover, with the endless possibilities in the combined area of ML and 2D materials, the advances covered herewith only represent the exciting start!

## Appendix

Table listing the full names of machine learning algorithms.

**Table:** 

Abbreviations	Full names	Abbreviations	Full names
ABR	Adaptive boosting regression	KMC	K‐means clustering
Adaboost	Adaptive boosting	KNR	K‐neighbors regression
ALIGNN	Atomistic line graph neural network	KRR	Kernel ridge regression
ANN	Artificial neural networks	LASSO	Least absolute shrinkage and selection operator
BNN	Bayesian neural network model	LGB	Light gradient boosting
BO	Bayesian optimization	LR	Linear regression
CCN	Concatenate convolutional networks	LSTM	Long short‐term memory
CGCNN	Crystal graph convolutional neural networks	MDC	Mean shift and density‐based spatial clustering
CLSTM	Convolutional long short‐term memory network	ML	Machine learning
CNN	Convolutional neural network	MLIPs	Machine‐learning interatomic potentials
DenseNet	Dense convolutional network	MLP	Multi‐layer perceptron
DL	Deep learning	MLR	Multiple linear regression
DNN	Deep neural network	MS	Mean shift
DT	Decision trees	PUML	positive and unlabeled machine learning
EA	Evolutionary algorithm	RCN	Residual convolutional networks
EL	Ensemble Learning	RCNN	Region convolutional neural network
ERT	Ensemble of regression tree	RF	Random forest
ET	Ensemble trees	RFE	Recursive feature elimination
ETR	Extra tree regression	RFR	Random forest regression
FCN	Fully convolutional network	RL	Reinforcement learning
FFNN	Feedforward neural network	RT	Regression tree
GA	Genetic algorithm	RVM	Relevance vector machine
GAP	Gaussian approximation potential	SGDC	Stochastic gradient descent classifier
GB	Gradient boosting	SISSO	Sure independence screening and sparsifying operator
GBDT	Gradient boosted decision tree	SVC	Support vector classifier
GBR	Gradient boosted regression	SVM	Support vector machine
GCN	Graph convolution networks	SVR	Support vector regression
GEP	Gene expression programming	TL	Transfer Learning
GGA	Generalized gradient approximation	TMINN	Transition‐metal interlink neural network
GNN	Graph neural network	UMLBC	unsupervised machine learning bilayer clustering
GNBC	Gaussian naive Bayes classification	VCN	VGG16 convolutional networks
GPR	Gaussian process regression	VDNNs	Voxel deep neural networks
KNN	K‐nearest neighbor	XGBoost	Extreme gradient boosting

## Conflict of Interest

The authors declare no conflict of interest.
